# Efficacy and safety of lipoprotein(a)-targeted therapeutics: a systematic review and network meta-analysis

**DOI:** 10.3389/fcvm.2026.1758366

**Published:** 2026-03-05

**Authors:** Jiaqiang Hu, Jun Wang, Haixia Zhang, Yaxi Jiang, Lihua Deng, Enwu Long, Song Liu

**Affiliations:** 1 Department of Pharmacy, Guang’an People's Hospital, Guang’an, Sichuan, China; 2 Affiliated Hospital of Zunyi Medical University, Zunyi, China; 3 Sichuan Province Key Laboratory of Personalized Drug Therapy, Sichuan Academy of Medical Sciences and Sichuan Provincial People's Hospital, China; 4 Department of Pharmacy, The Affiliated Hospital, Southwest Medical University, Luzhou, Sichuan, China

**Keywords:** antisense oligonucleotides, lipoprotein(a), lipoprotein(a)-targeted therapies, Muvalaplin, network meta-analysis, small interfering RNA

## Abstract

**Background:**

Lipoprotein(a)–targeted therapies are emerging approaches for lowering lipoprotein(a) [lp(a)].

**Objective:**

We conducted a systematic review and network meta-analysis to evaluate the efficacy and safety of lipoprotein(a)–targeted therapies in patients.

**Methods:**

We searched PubMed, Embase, Web of Science, and the Cochrane Central Register of Controlled Trials (CENTRAL) up to May 6, 2025, for randomized controlled trials (RCTs) with intervention duration of at least 12 weeks. The primary outcomes were percentage and absolute changes in Lp(a). Secondary outcomes included changes in low-density lipoprotein cholesterol (LDL-C) and apolipoprotein B (apoB), and safety outcomes including adverse events (AEs), serious adverse events (SAEs), and injection-site reactions. A frequentist framework network meta- analysis was performed.

**Results:**

Nine studies involving 1,432 participants were included. All six Lp(a)-targeted therapies significantly reduced Lp(a) levels. Compared with placebo, Olpasiran was the most effective therapy for both percentage [mean difference: −92.06, 95% (−109.80; −74.32), *P*-score: 0.94] and absolute reductions [−250.70 (−262.04; −239.36), *P*-score: 0.99], followed by Zerlasiran [−78.33 (−92.18; −64.48), *P*-score: 0.70], [−205.63 (−217.24; −194.03), *P*-score: 0.76]. In between-drug comparisons, Olpasiran was superior to Pelacarsen. Both Olpasiran and Zerlasiran were associated with improved LDL-C and apoB concentrations. Zerlasiran, Lepodisiran, and Pelacarsen were found to increase the risk of injection-site reactions.

**Conclusions:**

Lp(a)-targeted therapies achieved substantial reductions in Lp(a). Olpasiran was the most effective agent in lowering Lp(a) levels. These therapies also improved LDL-C and apoB. The majority of Lp(a)-targeted therapies demonstrate generally favorable safety profiles; However, injection-site reactions, particularly with Zerlasiran, warrant careful consideration.

**Systematic Review Registration:**

https://www.crd.york.ac.uk/PROSPERO/view/CRD420251069288, PROSPERO CRD420251069288.

## Introduction

Atherosclerotic cardiovascular disease (ASCVD) remains one of the leading causes of mortality worldwide and imposes a considerable economic burden on healthcare systems (1). Lp(a) has been identified as a significant contributor to residual cardiovascular risk in patients with ASCVD. Lp(a) promotes ASCVD progression through atherosclerosis, inflammation, thrombosis, and vascular calcification (2, 3), and is robustly associated with aortic valve calcification, stenosis, and adverse cardiovascular outcomes (4). Approximately 20%–30% of the global population—around two billion people—exhibit elevated Lp(a) levels, underscoring a significant unmet need for effective therapies aimed at lowering Lp(a) (5).

Structurally, Lp(a) consists of apolipoprotein(a) [apo(a)] covalently linked to apolipoprotein B-100 (apoB-100) on a low-density lipoprotein (LDL)-like particle and serves as a principal carrier of oxidized phospholipids (OxPL) (6). Plasma Lp(a) concentrations are highly heritable and largely determined by variation in the APOA gene (7). Studies indicate that on a per-particle basis, Lp(a) confers 5- to 6-fold greater atherogenic risk than LDL (8, 9).

Currently, there are no pharmacotherapies specifically approved for the reduction of Lp(a) levels. Statins remain the cornerstone of dyslipidemia management and are recommended for patients with elevated Lp(a) due to their significant reductions in overall cardiovascular risk (10). However, statins have no lowering effect on Lp(a) concentrations and may even contribute to an increase in these levels (11). While primarily developed for LDL-C reduction, proprotein convertase subtilisin/kexin type 9 (PCSK9) inhibitors, mipomersen, and ezetimibe have only a limited capacity to lower lipoprotein(a) levels (12). Genetic epidemiology suggests that substantial reductions in Lp(a) (approximately 85–250 nmol/L) may be required to achieve meaningful cardiovascular risk reduction (8, 13). Several small nucleic acid-based therapies and small molecules are currently under clinical investigation. Emerging therapies targeting *LPA* mRNA, including small interfering RNAs (siRNAs) and antisense oligonucleotides (ASOs), have shown substantial potential to markedly reduce Lp(a) by inducing the degradation of *LPA* mRNA, thereby inhibiting the synthesis of apo(a) protein and ultimately lowering Lp(a) levels. The oral small-molecule drug Muvalaplin inhibits Lp(a) formation by selectively blocking the covalent interaction between apo(a) and apoB-100. A Phase I clinical trial, 14 days of treatment with Muvalaplin reduced Lp(a) concentrations by up to 65%, underscoring its potential as a novel therapeutic strategy for Lp(a) lowering (14).

This systematic review and network meta-analysis aims to evaluate the efficacy and safety of emerging Lp(a)-targeted therapies, and to compare the advantages and disadvantages of different drug classes (siRNAs, ASOs, and small-molecule drugs), thereby providing robust evidence to inform the clinical management of elevated Lp(a).

## Methods

This meta-analysis was conducted and reported according to the Preferred Reporting Items for Systematic Reviews and Meta-Analysis (PRISMA) guidelines.The protocol for this meta-analysis was prospectively registered in the International Prospective Register of Systematic Reviews (PROSPERO; registration No. CRD420251069288).

### Search strategy

We searched PubMed, Embase, Web of Science, and Cochrane Central Register of Controlled Trials (CENTRAL) without language restrictions from database inception to 6 May 2025. Full details of the search strategy are available in (Supplementary Table S1).

### Eligibility criteria

We included all RCTs evaluating Lp(a)-targeted therapies vs. placebo or any comparator in adults (≥18 years). Eligible studies were required to report Lp(a)-related outcomes and have a minimum duration of 12 weeks, as this duration is generally considered sufficient for lipid parameters to reach a stable treatment effect. Two reviewers independently screened titles and abstracts, followed by full-text assessment of potentially eligible articles for final inclusion. Any disagreements were resolved through discussion with a third reviewer.

### Data extraction

Two reviewers independently extracted data using predefined standardized forms. Baseline characteristics such as age, sex, body mass index (BMI), and study duration were collected. The primary analysis evaluated the percentage and absolute changes in Lp(a) levels from baseline. Secondary outcomes included percentage changes in LDL-C and apoB levels, as well as safety outcomes, including adverse events, serious adverse events, and injection-site reactions. Any discrepancies in data extraction were resolved through discussion and consensus with a third investigator.

### Risk of bias assessment

Two reviewers independently assessed the risk of bias in included trials using the Cochrane Risk of Bias 2.0 (RoB 2) tool (15). Discrepancies were resolved by consultation with a third reviewer. Each trial was categorized as having low risk, some concerns, or high risk of bias according to RoB 2 criteria. The certainty of evidence was assessed using the confidence in network meta-analysis (CINeMA) framework (16). Publication bias was evaluated using funnel plots and Egger's regression test.

### Statistical analysis

Network meta-analyses were performed within a frequentist framework using random-effects models in R version 4.3.2 with the “netmeta” package. Mean differences (MDs) with 95% confidence intervals (CIs) were calculated for continuous outcomes, including Lp(a), LDL-C, and apoB levels. For dichotomous safety outcomes (local injection-site reactions, adverse events, and serious adverse events), odds ratios (ORs) with 95% CIs were estimated. The study also performed subgroup analyses to compare the efficacy and safety profiles across various classes of Lp(a)-lowering therapeutics, and further assessed interventions with different dosage levels and dosing intervals. Interventions were ranked based on efficacy and safety using *P*-scores. The *P*-score is a frequentist metric that quantifies the relative ranking of treatments and serves as a direct analogue to the Bayesian Surface Under the Cumulative Ranking curve (SUCRA) (17). A *P*-value <0.05 was considered statistically significant.

## Result

**Figure 1:**
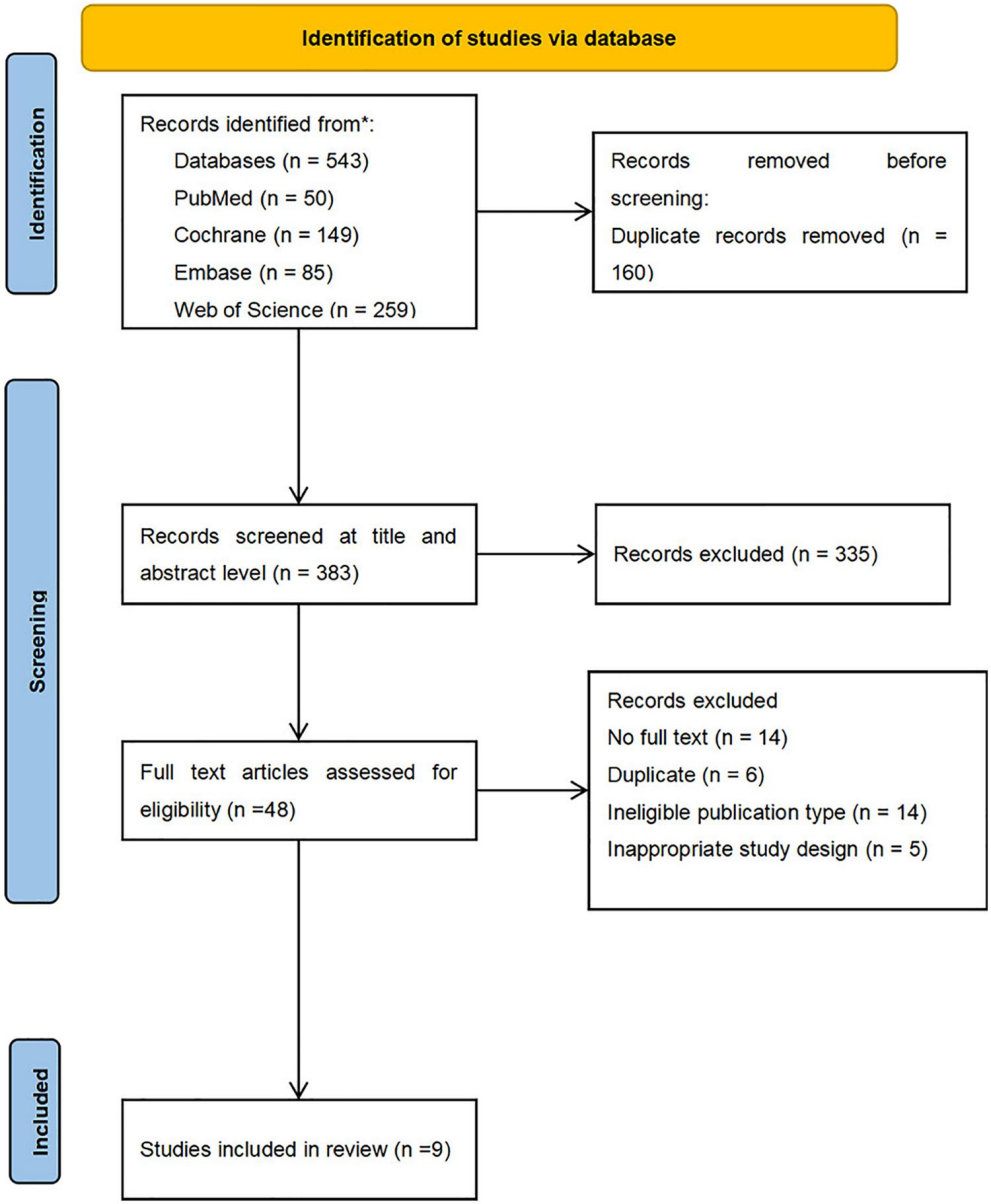
Flow chart for the identification, inclusion and exclusion of studies.

Initial systematic searches across PubMed, EMBASE, Web of Science, and Cochrane Library identified 543 records. After removal of 160 duplicates, 383 unique records were screened by title and abstract screening, resulting in the exclusion of 335 irrelevant records. After full-text assessment of 48 potentially eligible articles, 39 were excluded due to failure to meet the predefined inclusion criteria. Consequently, 9 articles were included in the meta-analysis (Figure 1).

Supplementary Table S2 summarizes study and participant characteristics. A total of 1,432 individuals were enrolled, investigating six Lp(a)-targeting agents [Pelacarsen, Olpasiran, Zerlasiran, Lepodisiran, Muvalaplin, and IONIS-APO(a) Rx]. Sample sizes ranged from 48 to 320, with study durations between 12 weeks (84 days) and 360 days. Details on study design, dosing regimens, administration frequency, age, sex, and baseline Lp(a) levels are presented in Supplementary Table S2.

### Risk of bias

Risk of bias for the change in Lp(a) was assessed as low in all trials. Publication bias was assessed using Egger's test and visual inspection of funnel plots. For all outcomes, Egger's test yielded non-significant results (*P* > 0.05), and the funnel plots did not reveal apparent asymmetry, suggesting no substantial evidence of publication bias (Supplementary Material). Nevertheless, given the relatively small number of included studies and the resulting limited statistical power, these findings should be interpreted with caution and considered exploratory (18) (Supplementary Figure S3).

### Lp(a) percentage reduction

**Figure 2:**
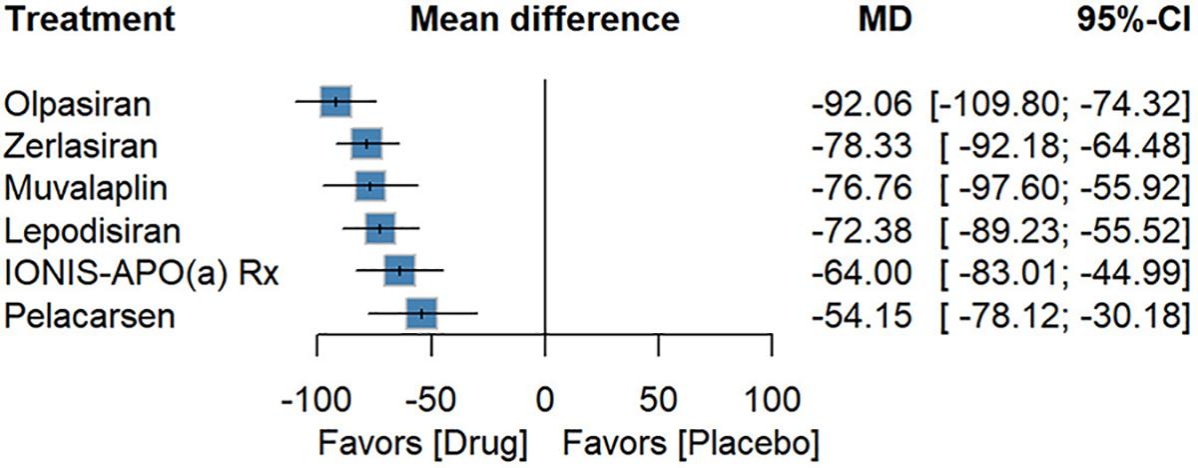
Forest plot for percentage reduction in Lp(a).

Eight RCTs (*n* = 1,432) reported percentage reductions in Lp(a). All six agents showed significant superiority over placebo (19–27). Olpasiran achieved the greatest effect [MD: −92.06, 95% (−109.80; −74.32), *P*-score: 0.94], followed by Zerlasiran [−78.33 (−92.18; −64.48), *P*-score: 0.70], Muvalaplin [−76.76 (−97.60; −55.92), *P*-score: 0.66], and Lepodisiran [−72.38 (−89.23; −55.52), *P*-score: 0.56]. IONIS-APO(a) Rx [−64.00 (−83.01; −44.99), *P*-score: 0.39] and Pelacarsen [−54.15 (−78.12; −30.18), *P*-score: 0.25] also achieved a reduction of more than 50% in Lp(a) levels(Figure 2). In between-drug comparisons, Olpasiran was superior to Pelacarsen [−37.91 (−67.73; −8.09)] (Supplementary Table S8.1).

### Lp(a) absolute reduction

**Figure 3:**
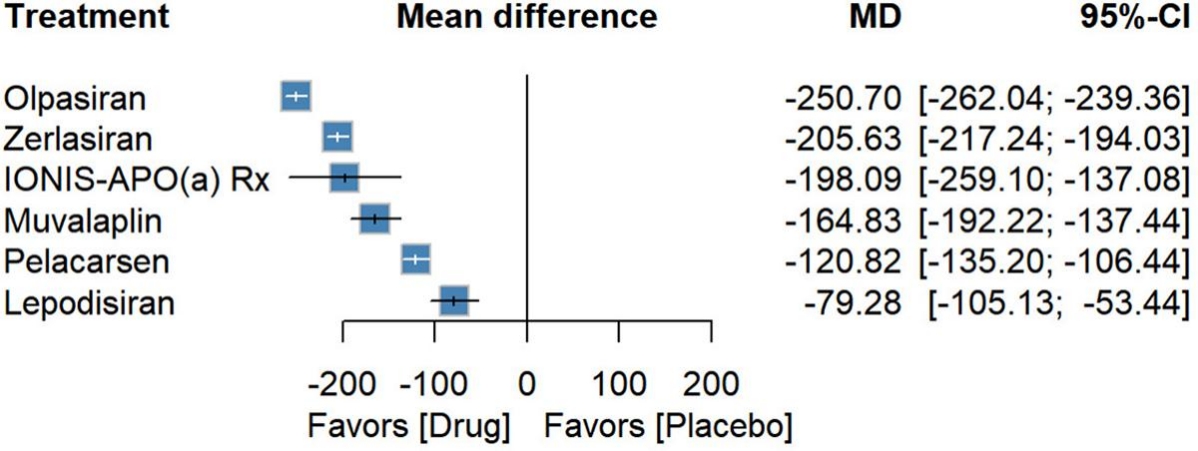
Forest plot for absolute reduction in Lp(a).

Six studies including 1,062 patients assessed absolute reductions in Lp(a). All five Lp(a)-targeted therapeutics were superior to placebo. Olpasiran yielded the largest reduction [−250.70 (−262.04; −239.36), *P*-score: 0.99], followed by Zerlasiran [−205.63 (−217.24; −194.03), *P*-score: 0.76] and IONIS-APO(a) Rx [−198.09 (−259.10; −137.08), *P*-score: 0.71]. Muvalaplin [−164.83 (–192.22 to −137.44), *P*-score: 0.53] and Pelacarsen [−120.82 (–135.20 to −106.44), *P*-score: 0.33] were also associated with significant reductions in Lp(a), and Lepodisiran [−79.28 (−105.13; −53.44), *P*-score: 0.17] was associated with comparatively smaller reductions (Figure 3). In between-drug comparisons, Olpasiran [−129.88 (−148.19; −111.57)], IONIS-APO(a) Rx [−77.27 (−139.95; −14.59)], and Muvalaplin [−44.01 (−74.94; −13.08)] demonstrated greater efficacy than Pelacarsen. IONIS-APO(a) Rx [−118.81 (−185.07; −52.55)] was more effective than Lepodisiran, and Olpasiran[−45.07 (−61.29; −28.84)] was superior to Zerlasiran (Supplementary Table S8.2).

### Effect on LDL-C reduction

**Figure 4:**
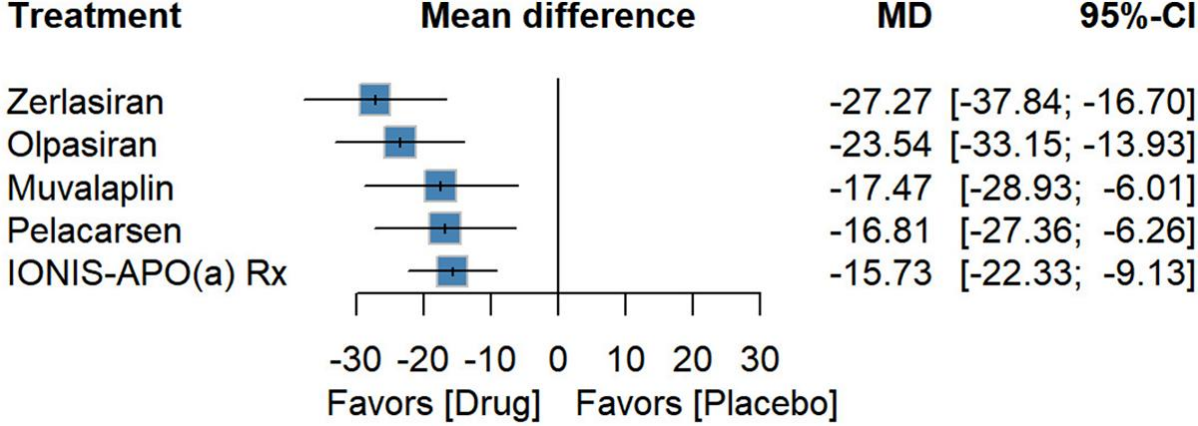
Forest plot for percentage reduction in LDL-C.

Six trials involving 1,064 participants were identified for assessing percentage changes in LDL-C. Compared with placebo, Zerlasiran produced the largest decrease [−27.27 (−37.84; −16.70), *P*-score: 0.89], followed by Olpasiran [−23.54 (−33.15; −13.93), *P*-score: 0.76], Muvalaplin [−17.47 (−28.93; −6.01), *P*-score: 0.49], Pelacarsen [−16.81 (−27.36; −6.26), *P*-score: 0.45], and IONIS-APO(a)Rx [−15.73 (−22.33; −9.13), *P*-score: 0.39] showed modest efficacy (Figure 4).

### Effect on ApoB reduction

**Figure 5:**
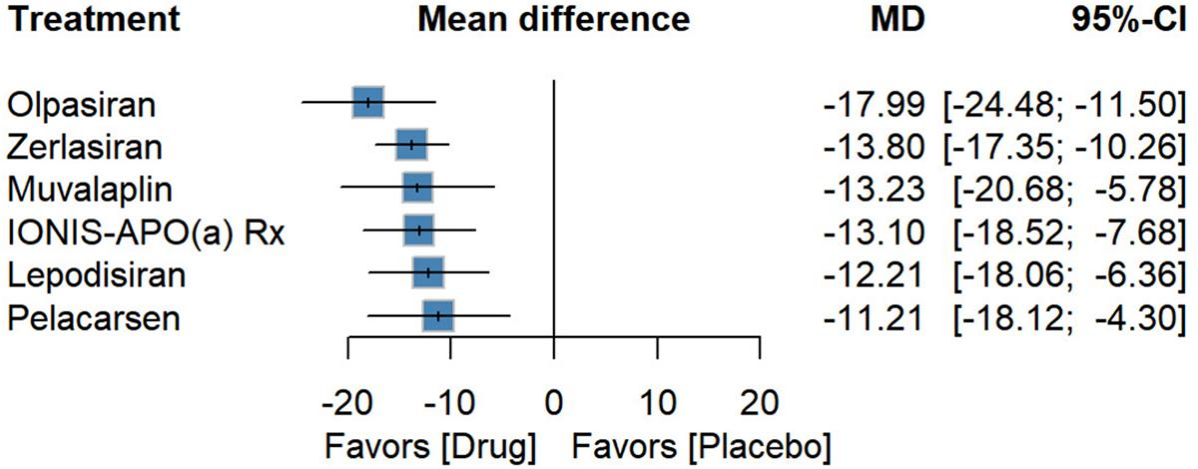
Forest plot for percentage reduction in apoB.

Compared with placebo, Olpasiran was most effective in lowering apoB [−17.99 (−24.48; −11.50), *P*-score: 0.90], followed by Zerlasiran [−13.80 (−17.35; −10.26), *P*-score: 0.62], Muvalaplin [−13.23 (−20.68; −5.78), *P*-score: 0.56], IONIS-APO(a) Rx[−13.10 (−18.52; −7.68), *P*-score: 0.55], Lepodisiran [−12.21 (−18.06; −6.36), *P*-score: 0.47], and Pelacarsen [−11.21 (−18.12; −4.30), *P*-score: 0.41] (Figure 5).

### Safety outcomes

**Figure 6:**
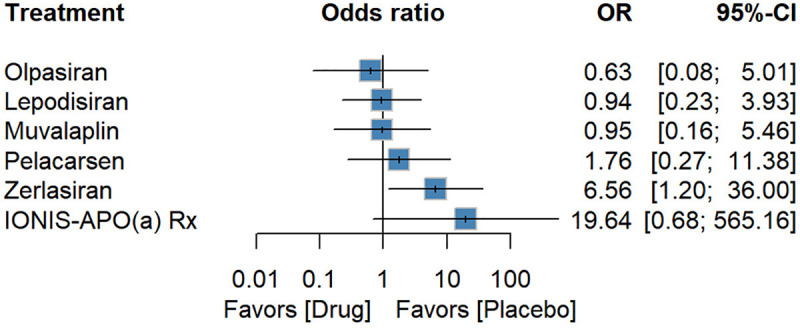
Forest plot for AEs.

**Figure 7:**
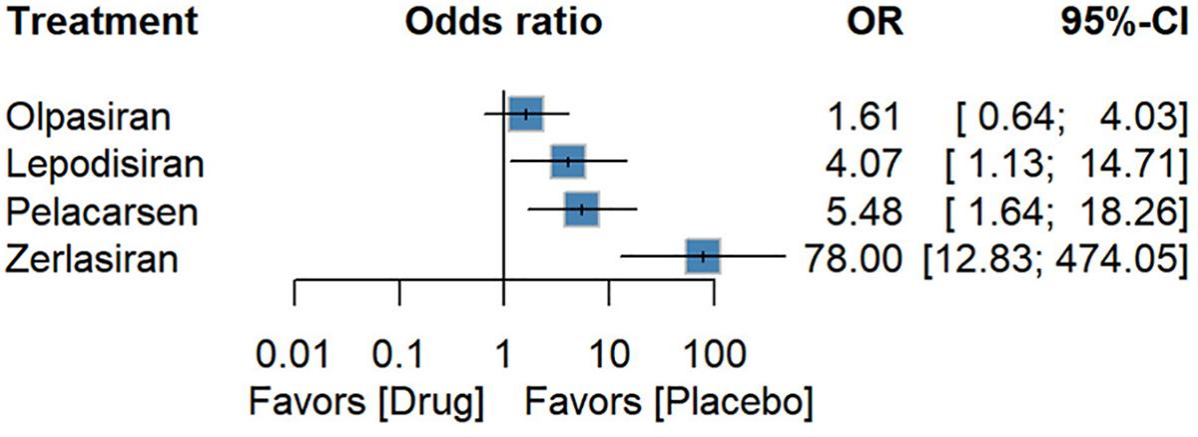
Forest plot for injection site reactions.

Eight RCTs reported adverse events, seven reported serious adverse events, and five reported injection-site reactions. Zerlasiran was associated with a higher risk of AEs compared with placebo [OR: 6.56 (1.20; 36.00)] (Figure 6). No other therapy showed significant differences in AEs or SAEs. For injection-site reactions, Olpasiran did not increase risk (OR 1.61, 0.64–4.03), whereas Lepodisiran [4.07, (1.13; 14.71), *P*-score: 0.44] and Pelacarsen [5.48, (1.64; 18.26), *P*-score: 0.36] were associated with higher risk. Zerlasiran showed a markedly elevated injection-site reactions risk [78.00 (12.83; 474.05), *P*-score: 0. 00] (Figure 7).

### Subgroup analyses

#### Subgroup analysis by drug class

**Figure 8:**
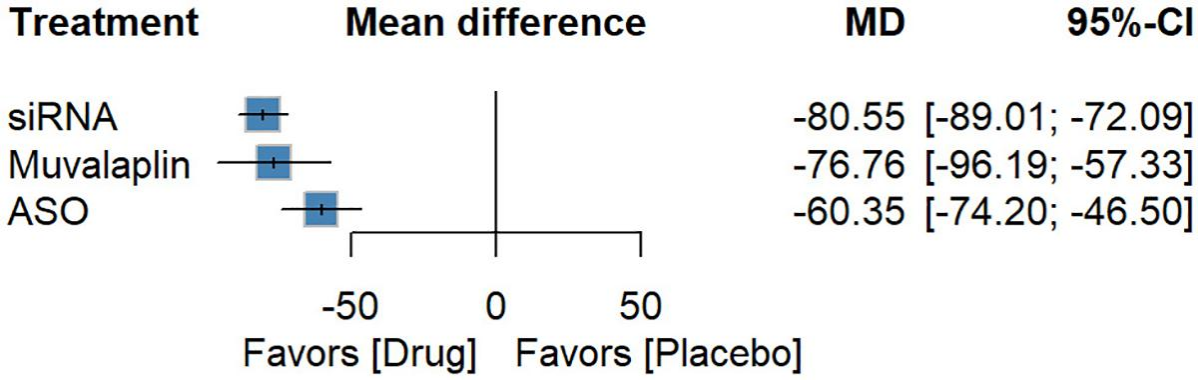
Forest plot for percentage reduction in different classes of Lp(a)-targeted therapies.

**Figure 9:**
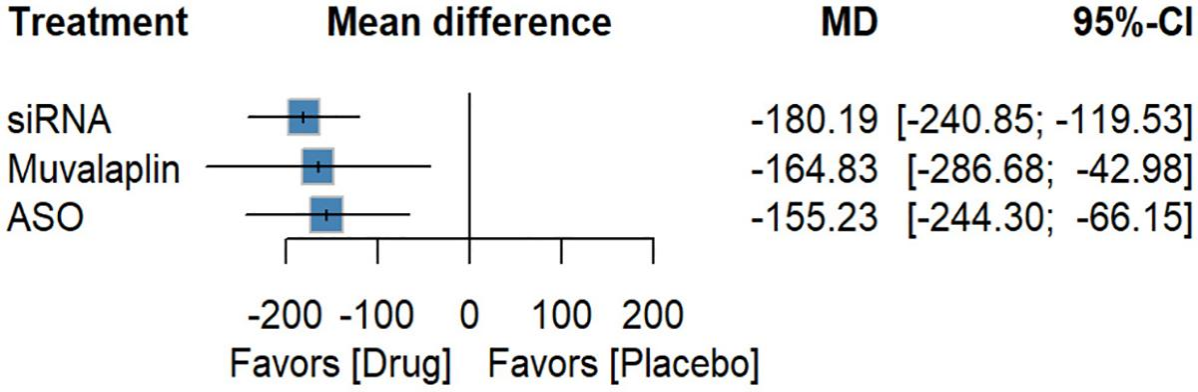
Forest plot for absolute reduction in different classes of Lp(a)-targeted therapies.

The Lp(a)-targeted drugs included ASOs, siRNAs, and the small molecule Muvalaplin. Subgroup analyses were conducted to explore potential differences in efficacy among these classes. Compared with placebo, siRNAs [−80.55 (−89.01; −72.09), *P*-score: 0.88], Muvalaplin [−76.76 (−96.19; −57.33), *P*-score: 0.76], and ASOs [−60.35 (−74.20; −46.50), *P*-score: 0.37] all substantially reduced Lp(a) percentage (Figure 8). siRNAs [−20.20 (−36.43; −3.97)] were more effective than ASOs. For absolute reductions, siRNAs [−180.19 (−240.85; −119.53), *P*-score: 0.75], Muvalaplin [−164.83 (−286.68; −42.98), *P*-score: 0.65], and ASOs [−155.23 (−244.30; −66.15), *P*-score: 0.59] all showed significant effects (Figure 9). In terms of safety, no significant differences were observed between any drug class and placebo with respect to overall AEs or SAEs (Supplementary Figure S7.4, Figure S7.5). However, siRNAs [6.35 (1.17 to 34.31)] were associated with an increased risk of injection-site reactions (Supplementary Figure S7.6).

#### Subgroup analyses of multiple doses

The included trials investigated Lp(a)-targeted agents across varying doses and administration frequencies. Subgroup analyses were performed to examine potential differences in efficacy by dosing regimen. Results indicated that Zerlasiran achieved the greatest reductions in both percentage and absolute Lp(a) levels at higher doses and with more frequent administration. Olpasiran also demonstrated robust efficacy at higher doses. Detailed findings are provided in the Supplementary Figure S7.7, Figure S7.8.

## Discussion

In this meta-analysis of eight RCTs of 1,432 patients, all Lp(a)-targeted therapies achieved substantial (>50%) reductions in Lp(a) levels compared with placebo, with additional benefits on LDL-C and apoB, and an overall acceptable safety profile. Olpasiran demonstrated the most consistent effects in lowering Lp(a) and apoB, while Zerlasiran was associated with the strongest LDL-C reduction but a higher risk of injection-site reactions. Moreover, siRNAs exhibited superior efficacy compared with ASOs in terms of percentage reductions in Lp(a).

This study provides the first comprehensive and systematic comparison of the efficacy and safety of different Lp(a)-targeted therapies. A prior network meta-analysis broadly assessed the impact of existing lipid-lowering agents on Lp(a) but did not include dedicated Lp(a)-targeted drugs (28). Another network meta-analysis evaluated siRNA therapies in patients with dyslipidemia and demonstrated the superiority of Lp(a)-specific agents over non-specific interventions (29); Our study advances previous evidence by systematically evaluating all classes of Lp(a)-targeted therapies, including siRNAs, ASOs, and Muvalaplin. Traditional lipid-lowering approaches aimed at Lp(a) have shown limited or unfavorable effects: statins may even increase Lp(a) (11); Niacin inhibits hepatic apo(a) expression (30), and the apoB antisense oligonucleotide mipomersen offered modest benefit but were limited by safety concerns (31), leading to discontinuation of Lp(a)-focused research. PCSK9 inhibitors and the CETP inhibitor obicetrapib have demonstrated only moderate reductions (32, 33). In contrast, Lp(a)-targeted therapies consistently achieved substantial lowering of Lp(a), highlighting their unique promise in addressing the residual cardiovascular risk attributable to elevated Lp(a).

Lp(a) is primarily synthesized in the liver. N-acetylgalactosamine (GalNAc), which specifically binds to the asialoglycoprotein receptor (ASGPR) on hepatocytes, enables enhanced hepatic targeting in therapeutics through conjugation to siRNA or ASO (34). In this study, IONIS-APO(a) Rx demonstrated a greater reduction in Lp(a) compared with placebo than Pelacarsen, which is largely attributable to its higher dosing. As a first-generation ASO, IONIS-APO(a) Rx lacks GalNAc modification, resulting in suboptimal delivery efficiency and potential accumulation in non-target organs. In contrast, Pelacarsen, the GalNAc-conjugated successor, exhibits 30-fold greater potency, enabling comparable Lp(a) lowering at significantly lower doses and less frequent administration (35).

Overall, siRNA therapies have shown superior potency and more sustained reductions in Lp(a) compared with ASOs. Both drug classes target the *LPA* gene but differ mechanistically: ASOs bind directly to *LPA* mRNA and promote RNA degradation, whereas siRNAs are incorporated into the RNA-induced silencing complex (RISC), where the antisense strand guides specific cleavage of *LPA* mRNA. The antisense strand within RISC is protected from degradation, and chemical modifications further enhance stability (36, 37). Moreover, RISC can repeatedly engage new mRNA molecules, conferring prolonged pharmacologic activity to siRNAs (38). Consequently, dosing frequency varies substantially across Lp(a)-targeted therapies. IONIS-APO(a) Rx requires weekly administration (27), the GalNAc-conjugated Pelacarsen allows monthly dosing, siRNA therapies can be administered every 12 weeks, and Olpasiran is being developed for dosing interval of up to 24 weeks (22). Notably, a single dose of lepodisiran has already demonstrated substantial Lp(a) reduction (21).

Despite the robust Lp(a)-lowering capacity of Lp(a)-targeted therapies, the clinical impact of these therapies on cardiovascular outcomes remains to be established in phase 3 outcome trials. Mendelian randomization studies suggest that an absolute reduction in Lp(a) of 125–215 nmol/L (approximately 60–100 mg/dL) confers cardiovascular benefit equivalent to lowering LDL-C by 38.7 mg/dL (13, 39). Our findings indicate that most Lp(a)-targeted agents have the potential to achieve this clinically meaningful threshold, with Olpasiran achieving the largest absolute reduction −250.7 [−262.04; −239.36] nmol/L. Although Lepodisiran produced the percentage reduction −72.38% [−89.23; −55.52], its absolute reduction [−79.28 (−105.13; −53.44) nmol/L] was limited by relatively low baseline Lp(a) levels in the study population. However, Lepodisiran exhibits dose-dependent effects, with a 96 mg dose achieving an absolute reduction of −155 nmol/L, approaching clinically meaningful thresholds (21). In trials enrolling individuals with higher baseline Lp(a) levels, a greater absolute reduction in Lp(a) would be expected (25). In addition, Pelacarsen, Olpasiran, and the oral small molecule Muvalaplin all reduce oxidized phospholipids (OxPL), a key proatherogenic factor, this effect may confer additional cardiovascular risk reduction. Ongoing phase 3 trials will be critical to confirm these findings (23, 26).

In terms of administration, both siRNA and ASO therapies require parenteral injection. In contrast, Muvalaplin offers the advantage of oral bioavailability, potentially improving patient adherence. Looking ahead, gene-editing strategies offer the promise of a one-time curative therapy. CTX320 is a CRISPR/Cas-based gene-editing therapy designed to selectively suppress LPA gene expression. In primary human hepatocytes, CTX320 achieves a dose-dependent reduction in Lp(a) levels exceeding 80% (40).

From a safety perspective, GalNAc conjugation not only improves hepatocyte targeting but also reduces systemic drug exposure, thereby lowering the risk of innate immune activation. Overall, Lp(a)-lowering therapies have shown favorable tolerability. Injection-site reactions have been observed with Zerlasiran, Lepodisiran, and Pelacarsen, with Zerlasiran most frequently associated. These injection-site reactions were generally transient and mild in severity, and did not lead to treatment discontinuation or missed doses (19). Therefore, these injection-site reactions are unlikely to affect patient adherence and are not expected to substantially influence clinical decision-making.

Ongoing Phase III trials targeting populations with elevated baseline Lp(a) levels complicated by ASCVD (NCT04023552, NCT05581303, NCT06292013) are expected to provide updated evidence on hard cardiovascular endpoints, which will be essential for evaluating the long-term clinical benefits of these therapies. Therefore, future updates on this topic will be needed with more studies available.

Our study has several limitations. First, the number of included trials and participants was relatively small, with most of the included studies being Phase I and Phase II trials, which may limit the robustness of the pooled estimates. Second, there is a critical lack of long-term cardiovascular outcome data. Nevertheless, multiple phase III trials of Lp(a)-targeted therapies are currently underway and are expected to provide more definitive data on efficacy, cardiovascular benefits, and safety. Third, heterogeneity in intervention doses and dosing intervals may have influenced the results, as Lp(a) reduction appears to be dose-dependent, potentially biasing the pooled estimates toward higher-dose regimens. Fourth, heterogeneity in baseline characteristics, particularly baseline Lp(a) levels, across the included studies may have influenced the pooled estimates. Among the nine included trials, most enrolled participants with markedly elevated baseline Lp(a) levels, whereas only one study included individuals with relatively lower baseline levels. Given the limited number of studies, subgroup analyses or meta-regression were not performed to formally adjust for baseline differences. Therefore, the potential impact of baseline heterogeneity should be considered when interpreting the results. Finally, due to the limited number of included studies, all trials were connected through placebo comparators, and no closed loops were formed, which limited formal assessment of network inconsistency.

In conclusion, Lp(a)-targeted therapies demonstrated substantial efficacy in reducing Lp(a) levels, with siRNA agents such as Olpasiran and Zerlasiran showing particularly promising results. In the absence of approved pharmacological options specifically designed to lower Lp(a), these agents highlight considerable therapeutic potential. Moreover, Lp(a)-targeted therapies were associated with improvements in apoB and LDL-C levelsLp(a)-targeted therapies exhibit a favorable safety profile, though attention should be given to injection-site reactions, particularly with Zerlasiran.

## Data Availability

The original contributions presented in the study are included in the article/Supplementary Material, further inquiries can be directed to the corresponding authors.
