# Supplementary material for: Efficacy and safety of lipoprotein(a)-targeted therapeutics: a systematic review and network meta-analysis
Source: Front Cardiovasc Med. 2026 Mar 5;13:1758366. doi: 10.3389/fcvm.2026.1758366 (PMC12999556; doi:10.3389/fcvm.2026.1758366)
Supplement: Supplementary file 1 [file Datasheet1.docx]

### Supplementary Material

**Efficacy and Safety of Lipoprotein(a)-Targeted Therapeutics: A systemic review and network meta-analysis**

**Table of contents**

[Supplementray Material 1: Search strategy 1](#_Toc27134)

[Supplementray Material 2: Characteristics of included studies 6](#_Toc22304)

[Supplementray Material 3: Risk of bias of randomized clinical trials 10](#_Toc4883)

[Supplementray Material 4: Evaluation of heterogeneity and inconsistency 11](#_Toc23652)

[Supplementray Material 5: Network maps 12](#_Toc13160)

[Supplementray Material 6: Funnel plots 19](#_Toc22052)

[Supplementray Material 7: forest plots of outcomes 26](#_Toc29834)

[Supplementray Material 8: League table of network meta-analysis results for Lp(a)-targeted therapeutics 30](#_Toc19380)

[Supplementray Material 9: P-scores of the effects of various Lp(a)-targeted therapeutics 35](#_Toc7313)

[Supplementray Material 10: CINeMA Results 37](#_Toc7835)

# Supplementray Material 1: Search strategy

**Table S1**. Search strategy

| PubMed |
| --- |
| #13 ((("Lipoprotein(a)"[Mesh]) OR (("Lipoprotein(a)"[Mesh]) OR (((Lipoprotein a[Title/Abstract]) OR (Lipoprotein Lp(a[Title/Abstract]))) OR (Lp(a[Title/Abstract]))))) AND (((("Oligonucleotides, Antisense"[Mesh]) OR ((((((antisense oligonucleotide[Title/Abstract]) OR (ASO[Title/Abstract])) OR (IONIS-APO(a)Rx[Title/Abstract])) OR (Pelacarsen[Title/Abstract])) OR (TQJ230[Title/Abstract])) OR (IONIS-APO(a)-LRx[Title/Abstract]))) OR (("RNA, Small Interfering"[Mesh]) OR ((((((((((((((small-interfering RNA[Title/Abstract]) OR (Short Interfering RNA[Title/Abstract])) OR (Interfering RNA, Small[Title/Abstract])) OR (siRNA[Title/Abstract])) OR (Interfering RNA, Short[Title/Abstract])) OR (Olpasiran[Title/Abstract])) OR (AMG 890[Title/Abstract])) OR (Lepodisiran[Title/Abstract])) OR (LY3819469[Title/Abstract])) OR (Zerlasiran[Title/Abstract])) OR (SLN360[Title/Abstract])) OR (Kylo-11[Title/Abstract])) OR (SRSD216[Title/Abstract])) OR (BW-20829[Title/Abstract])))) OR ((((Muvalaplin[Title/Abstract]) OR (LY3819469[Title/Abstract])) OR (HRS-5346[Title/Abstract])) OR (YS2302018[Title/Abstract])))) AND (((("Randomized Controlled Trials as Topic"[Mesh]) OR "Randomized Controlled Trial" [Publication Type]) OR (randomized controlled trial[Publication Type])) OR ((randomly[Text Word]) OR (clinical trial[Text Word])))  #12 ((("Randomized Controlled Trials as Topic"[Mesh]) OR "Randomized Controlled Trial" [Publication Type]) OR (randomized controlled trial[Publication Type])) OR ((randomly[Text Word]) OR (clinical trial[Text Word]))  #11 ((("Oligonucleotides, Antisense"[Mesh]) OR ((((((antisense oligonucleotide[Title/Abstract]) OR (ASO[Title/Abstract])) OR (IONIS-APO(a)Rx[Title/Abstract])) OR (Pelacarsen[Title/Abstract])) OR (TQJ230[Title/Abstract])) OR (IONIS-APO(a)-LRx[Title/Abstract]))) OR (("RNA, Small Interfering"[Mesh]) OR ((((((((((((((small-interfering RNA[Title/Abstract]) OR (Short Interfering RNA[Title/Abstract])) OR (Interfering RNA, Small[Title/Abstract])) OR (siRNA[Title/Abstract])) OR (Interfering RNA, Short[Title/Abstract])) OR (Olpasiran[Title/Abstract])) OR (AMG 890[Title/Abstract])) OR (Lepodisiran[Title/Abstract])) OR (LY3819469[Title/Abstract])) OR (Zerlasiran[Title/Abstract])) OR (SLN360[Title/Abstract])) OR (Kylo-11[Title/Abstract])) OR (SRSD216[Title/Abstract])) OR (BW-20829[Title/Abstract])))) OR ((((Muvalaplin[Title/Abstract]) OR (LY3819469[Title/Abstract])) OR (HRS-5346[Title/Abstract])) OR (YS2302018[Title/Abstract]))  #10 (((Muvalaplin[Title/Abstract]) OR (LY3819469[Title/Abstract])) OR (HRS-5346[Title/Abstract])) OR (YS2302018[Title/Abstract])  #9 ("RNA, Small Interfering"[Mesh]) OR ((((((((((((((small-interfering RNA[Title/Abstract]) OR (Short Interfering RNA[Title/Abstract])) OR (Interfering RNA, Small[Title/Abstract])) OR (siRNA[Title/Abstract])) OR (Interfering RNA, Short[Title/Abstract])) OR (Olpasiran[Title/Abstract])) OR (AMG 890[Title/Abstract])) OR (Lepodisiran[Title/Abstract])) OR (LY3819469[Title/Abstract])) OR (Zerlasiran[Title/Abstract])) OR (SLN360[Title/Abstract])) OR (Kylo-11[Title/Abstract])) OR (SRSD216[Title/Abstract])) OR (BW-20829[Title/Abstract]))  #8 (((((((((((((small-interfering RNA[Title/Abstract]) OR (Short Interfering RNA[Title/Abstract])) OR (Interfering RNA, Small[Title/Abstract])) OR (siRNA[Title/Abstract])) OR (Interfering RNA, Short[Title/Abstract])) OR (Olpasiran[Title/Abstract])) OR (AMG 890[Title/Abstract])) OR (Lepodisiran[Title/Abstract])) OR (LY3819469[Title/Abstract])) OR (Zerlasiran[Title/Abstract])) OR (SLN360[Title/Abstract])) OR (Kylo-11[Title/Abstract])) OR (SRSD216[Title/Abstract])) OR (BW-20829[Title/Abstract])  #7 "RNA, Small Interfering"[Mesh]  #6 ("Oligonucleotides, Antisense"[Mesh]) OR ((((((antisense oligonucleotide[Title/Abstract]) OR (ASO[Title/Abstract])) OR (IONIS-APO(a)Rx[Title/Abstract])) OR (Pelacarsen[Title/Abstract])) OR (TQJ230[Title/Abstract])) OR (IONIS-APO(a)-LRx[Title/Abstract]))  #5 (((((antisense oligonucleotide[Title/Abstract]) OR (ASO[Title/Abstract])) OR (IONIS-APO(a)Rx[Title/Abstract])) OR (Pelacarsen[Title/Abstract])) OR (TQJ230[Title/Abstract])) OR (IONIS-APO(a)-LRx[Title/Abstract])  #4 "Oligonucleotides, Antisense"[Mesh]  #3 ("Lipoprotein(a)"[Mesh]) OR (("Lipoprotein(a)"[Mesh]) OR (((Lipoprotein a[Title/Abstract]) OR (Lipoprotein Lp(a[Title/Abstract]))) OR (Lp(a[Title/Abstract]))))  #2 ("Lipoprotein(a)"[Mesh]) OR (((Lipoprotein a[Title/Abstract]) OR (Lipoprotein Lp(a[Title/Abstract]))) OR (Lp(a[Title/Abstract])))  #1 "Lipoprotein(a)"[Mesh] |
| Embase |
| #11 #3 AND #9 AND #10  #10 'controlled clinical trial'/exp OR 'controlled clinical trial' OR 'randomized controlled trial':ti,ab,kw OR 'clinical study':ti,ab,kw OR 'randomly':ti,ab,kw  #9 #4 OR #5 OR #6 OR #7 OR #8  #8 'muvalaplin':ti,ab,kw OR 'ly3819469':ti,ab,kw OR 'hrs-5346':ti,ab,kw OR 'ys2302018':ti,ab,kw  #7 'rna, small interfering':ti,ab,kw OR 'short interfering rna':ti,ab,kw OR 'sirna':ti,ab,kw OR 'sirnas':ti,ab,kw OR 'small interfering ribonucleic acid':ti,ab,kw OR 'small interfering rna':ti,ab,kw OR 'olpasiran':ti,ab,kw OR 'amg 890':ti,ab,kw OR 'lepodisiran':ti,ab,kw OR 'ly3819469':ti,ab,kw OR 'zerlasiran':ti,ab,kw OR 'sln360':ti,ab,kw OR 'kylo-11':ti,ab,kw OR 'srsd216':ti,ab,kw OR 'bw-20829':ti,ab,kw  #6 'small interfering rna'/exp  #5 'oligonucleotides, antisense':ti,ab,kw OR 'oligoribonucleotides, antisense':ti,ab,kw OR 'antisense oligonucleotide':ti,ab,kw OR 'aso':ti,ab,kw OR 'ionis-apo(a)rx':ti,ab,kw OR 'pelacarsen':ti,ab,kw OR 'tqj230':ti,ab,kw OR 'ionis-apo(a)-lrx':ti,ab,kw  #4 'antisense oligonucleotide'/exp  #3 #1 OR #2  #2 'lipoprotein(a)':ti,ab,kw OR 'lipoprotein a':ti,ab,kw OR 'lp(a)':ti,ab,kw OR 'lipoprotein lp(a)':ti,ab,kw  #1 'lipoprotein a'/exp |
| Cochrane Library |
| #1 MeSH descriptor: [Lipoprotein(a)] explode all trees  #2 (Lipoprotein (a)):ti,ab,kw OR (Lipoprotein Lp(a)):ti,ab,kw OR (Lipoprotein a):ti,ab,kw OR (Lp(a)):ti,ab,kw  #3 #1 OR #2  #4 MeSH descriptor: [Oligonucleotides, Antisense] explode all trees  #5 (antisense oligonucleotide):ti,ab,kw OR (ASO):ti,ab,kw OR (IONIS-APO(a)Rx):ti,ab,kw OR (Pelacarsen):ti,ab,kw OR (TQJ230):ti,ab,kw  #6 MeSH descriptor: [RNA, Small Interfering] explode all trees  #7 (small-interfering RNA):ti,ab,kw OR (Short Interfering RNA):ti,ab,kw OR (Interfering RNA, Small):ti,ab,kw OR (Interfering RNA, Short):ti,ab,kw OR (siRNA):ti,ab,kw OR (Olpasiran):ti,ab,kw OR (AMG 890):ti,ab,kw OR (Lepodisiran):ti,ab,kw OR (LY3819469):ti,ab,kw OR (Zerlasiran):ti,ab,kw OR (SLN360):ti,ab,kw OR (Kylo-11):ti,ab,kw OR (SRSD216):ti,ab,kw OR (BW-20829):ti,ab,kw  #8 (Muvalaplin):ti,ab,kw OR (LY3819469):ti,ab,kw OR (HRS-5346):ti,ab,kw OR (YS2302018):ti,ab,kw  #9 #4 OR #5 OR #6 OR #7 OR #8  #10 (Randomized Controlled Trial):pt OR (controlled clinical trial):pt OR (random*):ti,ab,kw OR (clinical trial):ti,ab,kw OR (randomly):ti,ab,kw (Word variations have been searched)  #11 #3 AND #9 AND #10 |
| Web of Science |
| #1 "((((((TS=(Oligonucleotides, Antisense)) OR TS=(antisense oligonucleotide)) OR TS=(ASO)) OR TS=(IONIS-APO(a)Rx)) OR TS=(Pelacarsen)) OR TS=(TQJ230)) OR TS=(IONIS-APO(a)-LRx) and Preprint Citation Index (Exclude – Database)"  #2 "((((((((((((((TS=(RNA, Small Interfering)) OR TS=(small-interfering RNA)) OR TS=(Short Interfering RNA)) OR TS=(Interfering RNA, Small)) OR TS=(Interfering RNA, Short)) OR TS=(siRNA)) OR TS=(Olpasiran)) OR TS=(AMG 890)) OR TS=(Lepodisiran)) OR TS=(LY3819469)) OR TS=(Zerlasiran)) OR TS=(SLN360)) OR TS=(Kylo-11)) OR TS=(SRSD216)) OR TS=(BW-20829) and Preprint Citation Index (Exclude – Database)"  #3 "(((TS=(Muvalaplin)) OR TS=(LY3819469)) OR TS=(HRS-5346)) OR TS=(YS2302018) and Preprint Citation Index (Exclude – Database)"  #4 "((((TS=(randomized controlled tria* )) OR TS=(controlled clinical tria*)) OR TS=(clinical tria*)) OR TS=(random* )) OR TS=(tria*) and Preprint Citation Index (Exclude – Database) "  #5 "((TS=(""Lipoprotein(a)"")) OR TS=(""Lipoprotein Lp(a)"")) OR TS=(""Lp(a) "") and Preprint Citation Index (Exclude – Database)"  #6 "#1 OR #2 OR #3 and Preprint Citation Index (Exclude – Database) "  #7 "#4 AND #5 AND #6 and Preprint Citation Index (Exclude – Database)" |

# Supplementray Material 2: Characteristics of included studies

**Table S2**: Baseline of characteristics of included studies

| **Study** | **NCT Number** | **Trial Phase** | **Race or Country** | **Follow-up duration** | **Number of participants** | **Randomized  treatments** | **Dose and frequency** | **age,  mean (SD),  years** | **Male n(%)** | **Lipoprotein(a)  nmol/l** | **LDL cholesterol  mg/dL** | **Apolipoprotein B ,mg/dL** |
| --- | --- | --- | --- | --- | --- | --- | --- | --- | --- | --- | --- | --- |
| Nissen  2024 | NCT04606602 | 1 | Asian, Black or  African American, White | 150 days | 36 | Zerlasiran:9 | 200 mg q4w | 59.8 (8.6) | 7 (77.8) | 266 (194-295)* | 69 (38.0) | 71 (34) |
|  |  |  |  |  |  | Zerlasiran:9 | 300 mg q8w sc | 61.3 (6.8) | 4 (44.4) | 316 (276-377)* | 56 (19.5) | 59 (14) |
|  |  |  |  |  |  | Zerlasiran:9 | 450 mg q8w sc | 56.9 (12.2) | 5 (55.6) | 247 (183-350)* | 77 (39.7) | 69 (23) |
|  |  |  |  |  |  | Placebo:9 |  | 47.0 (8.2) | 5 (55.6) | 292 (217-389)* | 67 (23.9) | 69 (23) |
| Nissen  2022 | NCT04606602 | 1 | Asian, Black or African American, White | 150 days | 32 | Zerlasiran:6 | 30 mg sc | 45.5 (10.5) | 4 (67) | 171 (142-219)* | 113 (38) | 83 (23) |
|  |  |  |  |  |  | Zerlasiran:6 | 100 mg sc | 46.3 (12.3) | 4 (67) | 217 (202-274)* | 121 (46) | 94 (29) |
|  |  |  |  |  |  | Zerlasiran:6 | 300 mg sc | 58.7 (13.2) | 2 (33) | 285 (195-338)* | 100 (25) | 89 (6) |
|  |  |  |  |  |  | Zerlasiran:6 | 600 mg sc | 43.7 (17.5) | 3 (50) | 231 (179-276)* | 108 (54) | 81 (25) |
|  |  |  |  |  |  | Placebo:8 |  | 52.9 (12.0) | 2 (25) | 238 (203-308)* | 99 (48) | 81 (30) |
| Nissen  2023 | NCT04914546 | 1 | Asian, Black or African American,  Hispanic or Latino, Non-Hispanic,White | 48 weeks | 48 | Lepodisiran:6 | 4 mg sc | 40.5 (11.7) | 5 (83.3) | 78 (50-152)* | 108 (34) | 90 (22) |
|  |  |  |  |  |  | Lepodisiran:6 | 12 mg sc | 44.3 (8.3) | 5 (83.3) | 97  (86-107)* | 148 (38) | 117 (38) |
|  |  |  |  |  |  | Lepodisiran:6 | 32 mg sc | 50.7 (11.3) | 3 (50.0) | 120 (110-188)* | 110 (13) | 93 (13) |
|  |  |  |  |  |  | Lepodisiran:6 | 96 mg sc | 47.8 (10.4) | 3 (50.0) | 167 (124-189)* | 118 (17) | 97 (17) |
|  |  |  |  |  |  | Lepodisiran:6 | 304 mg sc | 51.8 (9.5) | 4 (66.7) | 96  (72-132)* | 142 (27) | 114 (22) |
|  |  |  |  |  |  | Lepodisiran:6 | 608 mg sc | 38.5 (15.6) | 5 (83.3) | 130  (87-151)* | 135 (31) | 108 (32) |
|  |  |  |  |  |  | Placebo:12 |  | 50.3 (11.1) | 6 (50.0) | 111  (78-134)* | 143 (36) | 123 (30) |
| O'Donoghue2022 | NCT04270760 | 2 | North America,  Europe, Australia, Japan | 96 weeks | 281 | Placebo:54 |  | 63.4(8.9) | 36 (67) | 246.1  (199.9-343.3)* | 64.8  (47.5-81.0)* | 62.5 (48.5-76.0)* |
|  |  |  |  |  |  | Olpasiran:58 | 10 mg q12w sc | 63.4(9.5) | 46 (79) | 304.0 (194.2-397.6)* | 69.0 (52.0-83.5)* | 66.8 (51.5-81.5)* |
|  |  |  |  |  |  | Olpasiran:58 | 75 mg q12w sc | 61.3(9.2) | 35 (60) | 227.5 (188.4-304.2)* | 75.0 (53.5-90.0)* | 74.0 (59.5-85.0)* |
|  |  |  |  |  |  | Olpasiran:56 | 225 mg q12w sc | 59.7(10.1) | 41 (73) | 265.4 (200.6-342.2)* | 62.3 (48.5-80.5)* | 65.8 (49.5-80.8)* |
|  |  |  |  |  |  | Olpasiran:55 | 225 mg q24w sc | 61.8(9.4) | 33 (60) | 283.4 (204.6-389.2)* | 62.3 (48.5-80.6)* | 64.0 (56.5-79.0)* |
| Nissen  2024a | NCT05537571 | 2 | Asian, Black, White | 36 weeks | 178 | Zerlasiran:45 | 450 mg q24w sc | 63.8 (10.2) | 34 (75.6) | 216 (182-337)* | 71 (33) | 75 (23) |
|  |  |  |  |  |  | Zerlasiran:42 | 300 mg q16w sc | 63.1 (9.8) | 31 (73.8) | 221 (181-270)* | 69 (35) | 77 (30) |
|  |  |  |  |  |  | Zerlasiran:44 | 300 mg q24w sc | 64.5 (8.7) | 31 (70.5) | 225 (183-296)* | 69 (30) | 75 (22) |
|  |  |  |  |  |  | Placebo: 47 |  | 63.6 (9.1) | 36 (76.6) | 203 (164-242)* | 64 (28) | 72 (19) |
| Tsimikas  2020 | NCT03070782 | 2 | White | 25 weeks | 286 | pelacarsen: 48 | 20 mg q4w sc | 60.0(9.6) |  | 246.6  (179.2–300.8)* | 89.3(37.1) | 80.7(23.6) |
|  |  |  |  | 25 weeks |  | pelacarsen: 48 | 40 mg q4w sc | 61.3(10.6) |  | 220.0  (176.5–283.3)* | 77.4(39.5) | 71.9(23.4) |
|  |  |  |  | 27 weeks |  | pelacarsen: 48 | 20 mg q2w sc | 57.9(11.5) |  | 238.2  (183.7–298.4)* | 74.4(28.8) | 69.3(19.8) |
|  |  |  |  | 25 weeks |  | pelacarsen: 47 | 60 mg q4w sc | 62.2(9.7) |  | 204.5  (163.8–286.5)* | 67.6(28.3) | 68.5(18.8) |
|  |  |  |  | 27 weeks |  | pelacarsen: 48 | 20 mg qw sc | 58.9(8.0) |  | 233.7  (193.1–275.3)* | 76.1(28.4) | 70.6(19.2) |
|  |  |  |  | 25 weeks or  27 weeks |  | Placebo: 47 |  | 59.9(10.5) |  | 231.6  (194.9–317.7)* | 79.4(29.2) | 73.8(16.9) |
| Viney  2016 | NCT02160899 | 2 | White, Black, Other Race | 12weeks | 61 | Placebo: 26 |  | 54 (10) | 20 (77) | 251.6 (81.5) | 124.0 (42.7) | 100.2 (28.3) |
|  |  |  |  |  | cohort A | IONIS-APO(a) Rx: 24 | 100-300mg qw sc  (Dose escalated every 4 weeks) | 55 (7) | 11 (46) | 254.4 (81.8) | 127.7 (44.5) | 99.4 (24.8) |
|  |  |  |  |  | cohort B | Placebo: 3 |  | 62 (8) | 0 | 488.3 (59.7) | 87.2 (16.3) | 85.2 (5.3) |
|  |  |  |  |  |  | IONIS-APO(a) Rx: 8 | 100-300mg qw sc  (Dose escalated every 4 weeks) | 61 (8 | 2 (25) | 444.9 (105.3) | 111.8 (38.9) | 94.6 (22.5) |
| Nicholls  2025 | NCT05563246 | 2 | Asian, Black, HispanicorLatino, Native Hawaiian or Other PacificIslander, White, Multiple | 12 weeks | 233 | Muvalaplin: 34 | 10mg qd po | 66.5(58.0-71.0)* | 23(67.6) | 211.0(93.53) | 78.0 (36.66): | 79.5(24.25) |
|  |  |  |  |  |  | Muvalaplin: 64 | 60mg qd po | 67.0(59.5-71.0)* | 42(65.6) | 198.2(89.98) | 70.3 (33.67): | 74.2(23.04) |
|  |  |  |  |  |  | Muvalaplin: 68 | 240mg qd po | 66.0(58.0-70.5)* | 44(64.7) | 223.5(101.54) | 69.7 (33.29): | 77.3(24.02) |
|  |  |  |  |  |  | Placebo: 67 |  | 63.0(55.0-70.0)* | 48(71.6) | 234.2(106.66) | 70.7 (33.82) | 75.5(23.37) |
| Nissen  2025 | NCT05565742 | 2 | White, Black, Asian, Multiracial or mixed race, Hispanic or Latino | 360 days | 320 | Placebo:69 |  | 63.5(8.4) | 37 (54) | 241.9  (202.9–301.7)* |  | 75.0  (63.0–93.0)* |
|  |  |  |  |  |  | Lepodisiran:36 | 16 mg q180d sc | 62.6(10.6) | 27 (75) | 243.2  (203.4–313.3)* |  | 82.5  (67.0–94.5)* |
|  |  |  |  |  |  | Lepodisiran:74 | 96 mg q180d sc | 63.8(9.9) | 39 (53) | 262.0  (213.0–325.3)* |  | 83.0  (67.0–101.0)* |
|  |  |  |  |  |  | Lepodisiran:72 | 400 mg sc | 62.2(9.7) | 45 (62) | 264.1  (201.1–331.1)* |  | 79.0  (65.5–91.0)* |
|  |  |  |  |  |  | Lepodisiran:69 | 400 mg q180d sc | 61.4(10.9) | 34 (49) | 242.2  (199.7–329.9)* |  | 79.0  (69.0–97.0)* |

Data are presented as mean (SD).

*: Median (interquartile range, IQR);

# Supplementray Material 3: Risk of bias of randomized clinical trials

**Figure S3**: Overall risk of bias presented as percentage of each risk of bias item across all included studies.

Green = Low risk, Red = High risk, Yellow = Some concerns.


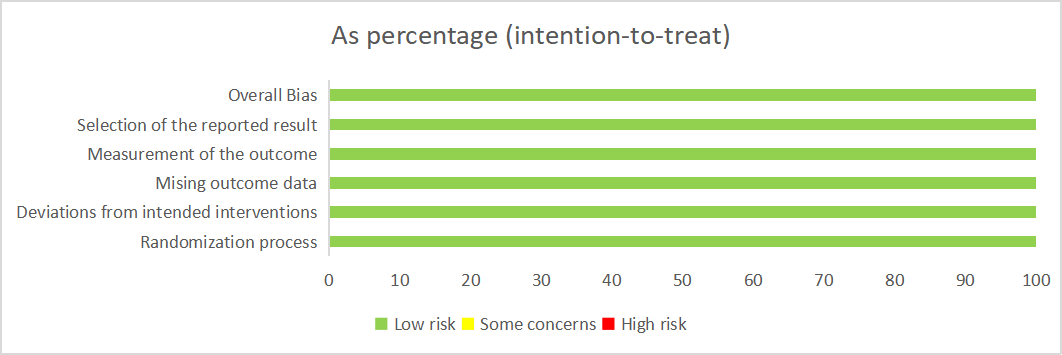


Table S3: Study level risk of bias assessment using Cochrane risk of bias tool 2.0 for assessing risk of bias of randomized clinical trials.

| **Unique ID** | **Study ID** | **Randomization process** | **Deviations from intended**  **interventions** | **Mising outcome data** | **Measurement**  **of the outcome** | **Selection of the reported result** | **Overall Bias** |
| --- | --- | --- | --- | --- | --- | --- | --- |
| Nissen2024 | NCT04606602 | Low | Low | Low | Low | Low | Low |
| Nissen2022 | NCT04606602 | Low | Low | Low | Low | Low | Low |
| Nissen2023 | NCT04914546 | Low | Low | Low | Low | Low | Low |
| O'Donoghue  2022 | NCT04270760 | Low | Low | Low | Low | Low | Low |
| Tsimikas2020 | NCT03070782 | Low | Low | Low | Low | Low | Low |
| Nissen2024a | NCT05537571 | Low | Low | Low | Low | Low | Low |
| Nissen2025 | NCT05565742 | Low | Low | Low | Low | Low | Low |
| Nicholls2025 | NCT05563246 | Low | Low | Low | Low | Low | Low |
| Viney2016 | NCT02160899 | Low | Low | Low | Low | Low | Low |

# Supplementray Material 4: Evaluation of heterogeneity and inconsistency

| **Outcome** | **Estimated τ^2^** | **I^2^** | **Q** | **p-value** |
| --- | --- | --- | --- | --- |
| Lp(a) percentage reduction | 77.1093 | 65.80% | 5.85 | 0.0536 |
| Lp(a) absolute reduction | 188.4191 | 44.80% | 1.81 | 0.1785 |
| LDL-C | 0 | 0% | 0.05 | 0.8225 |
| Apo-B | 0.6865 | 7.20% | 1.08 | 0.2993 |
| Adverse events | 0.7136 | 54.60% | 4.41 | 0.1105 |
| Serious adverse events | 0 | 0% | 0.02 | 0.8801 |
| Injection-site reaction | 0 | 0% | 0.21 | 0.6452 |
| Subgroup Analysis by drug class | | | | |
| Lp(a): % | 62.3318 | 78.90% | 23.72 | 0.0002 |
| Lp(a): absolute reduction | 3669.7272 | 97.40% | 154.17 | < 0.0001 |
| LDL-C | 0 | 0% | 0.34 | 0.9522 |
| Apo-B | 0 | 0% | 3.06 | 0.548 |
| Adverse events | 1.4672 | 75.40% | 20.33 | 0.0011 |
| Serious adverse events | 0 | 0% | 1.05 | 0.9026 |
| Injection-site reaction | 2.2859 | 79.10% | 14.36 | 0.0025 |

# Supplementray Material 5: Network maps

**Figure S5.1**: Network map of Percentage Reduction in Lp(a)


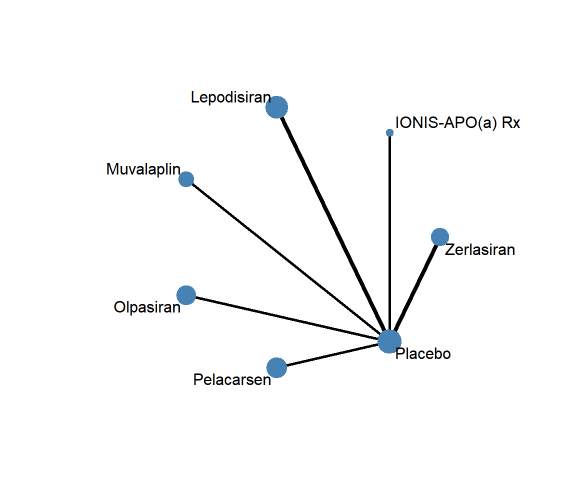


**Figure S5.2:** Network map of absolute reduction in Lp(a)


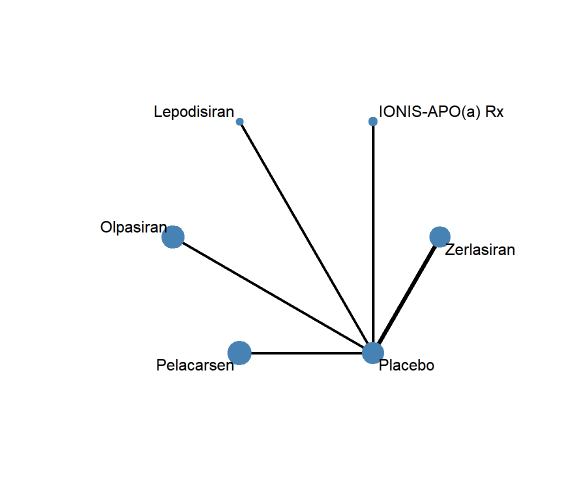


**Figure S5.3**: Network map of LDL-C


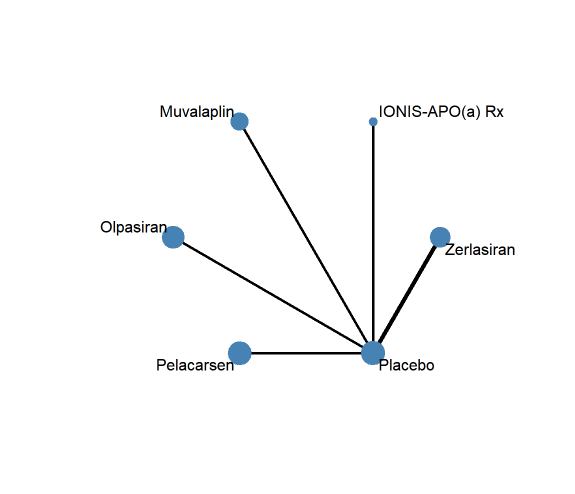


**Figure S5.4**: Network map of ApoB


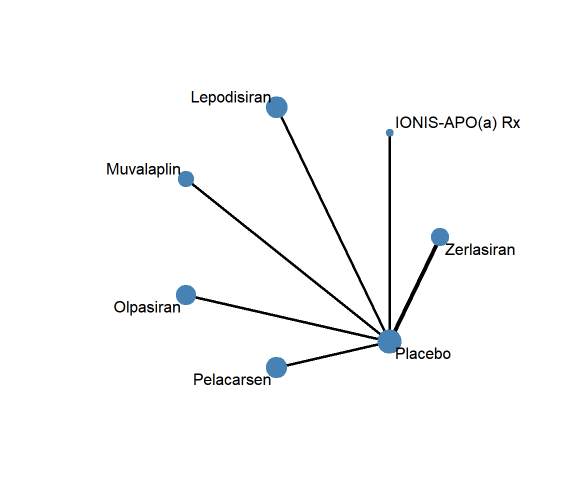


**Figure S5.5**: Network map of AEs


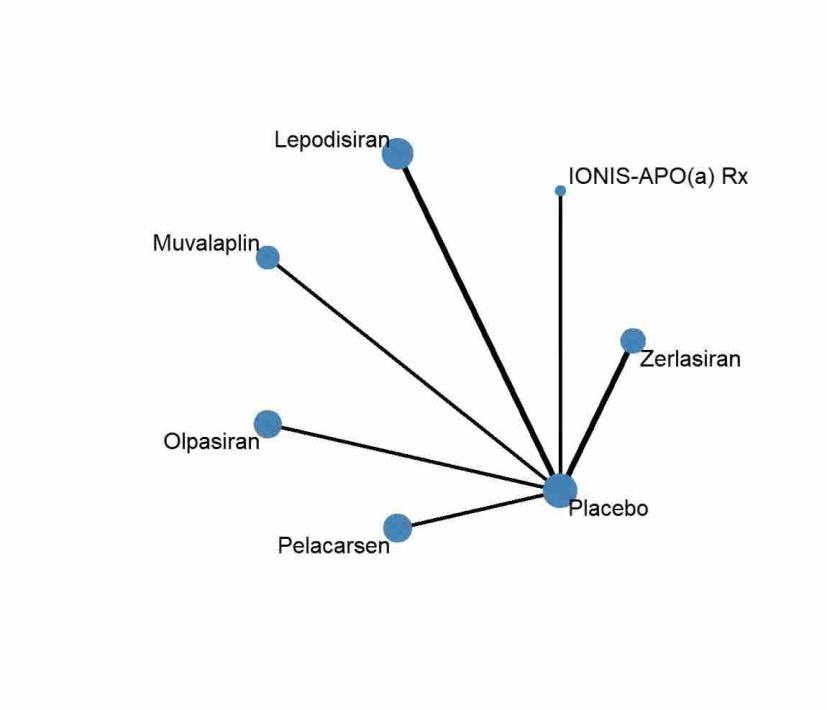


**Figure S5.6**: Network map of SAEs


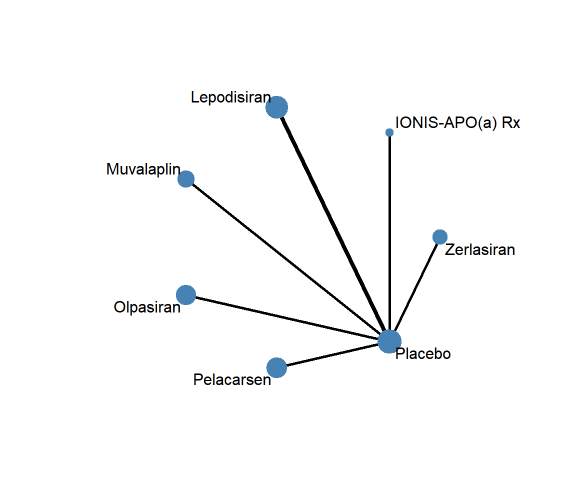


**Figure S5.7**: Network map of Injection site reactions


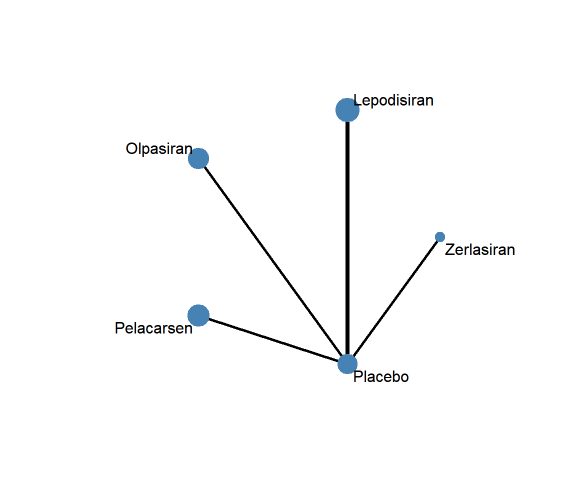


**Figure S5.8**: Network map of Lp(a) Percentage Reduction by drug class


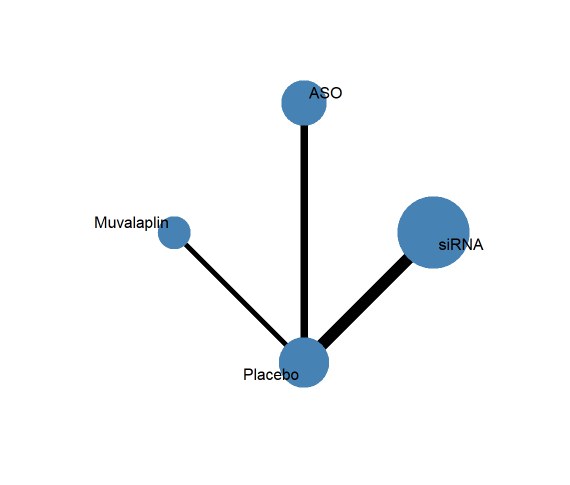


**Figure S5.9**: Network map of Lp(a) Absolute Reduction by drug class


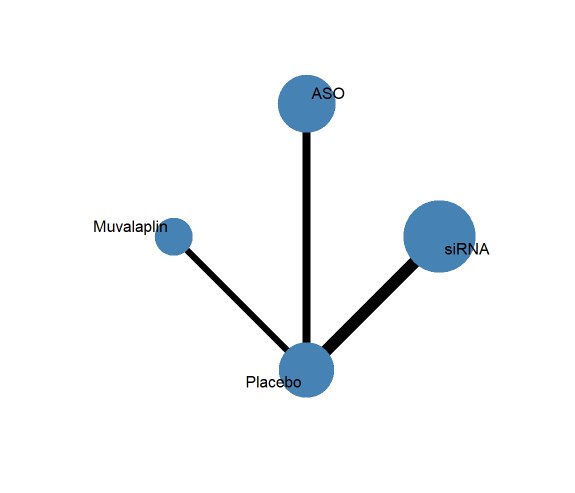


**Figure S5.10**: Network map of LDL-C by drug class


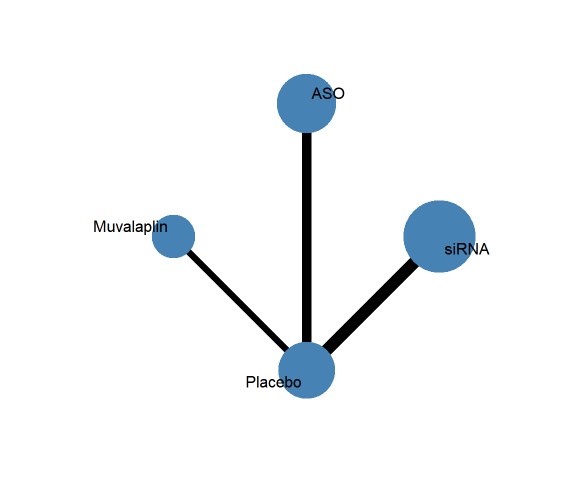


**Figure S5.11**: Network map of ApoB by drug class


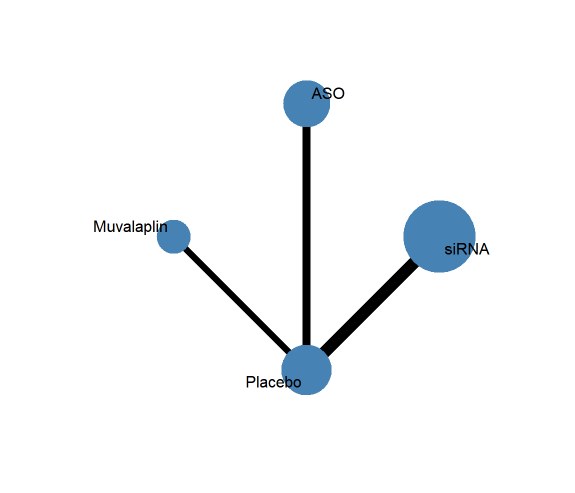


**Figure S5.12**: Network map of AEs by drug class


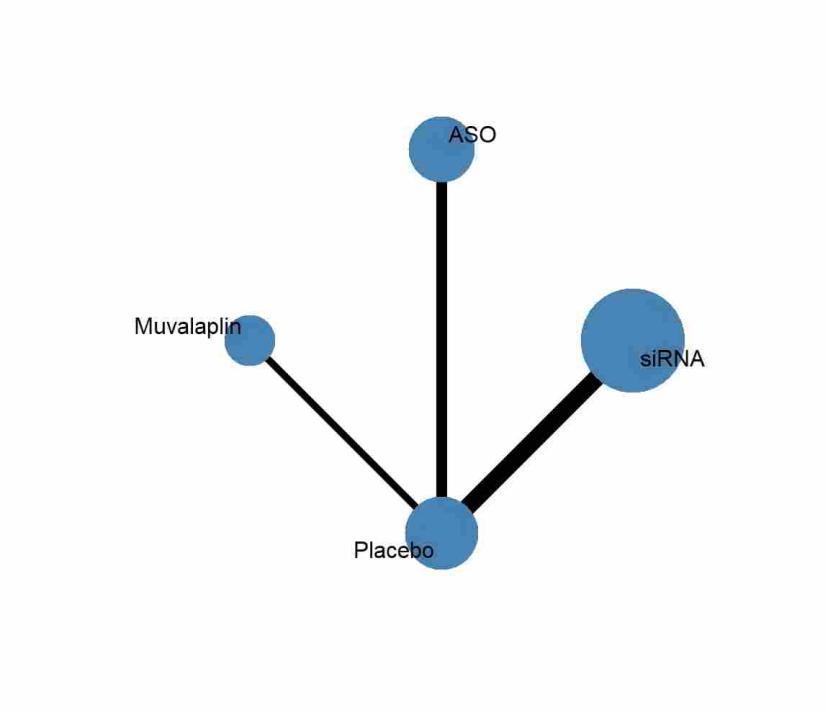


**Figure S5.13**: Network map of SAEs by drug class


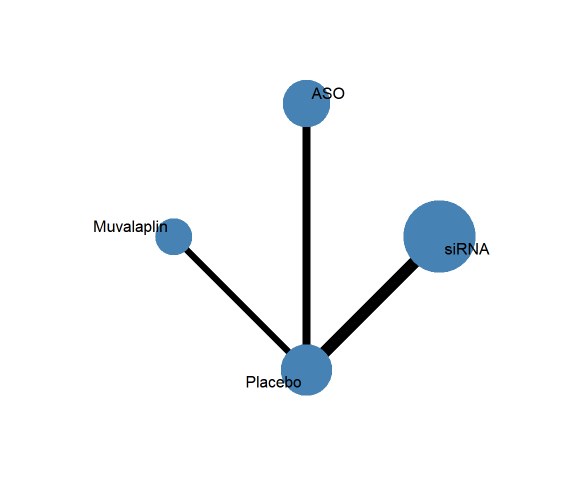

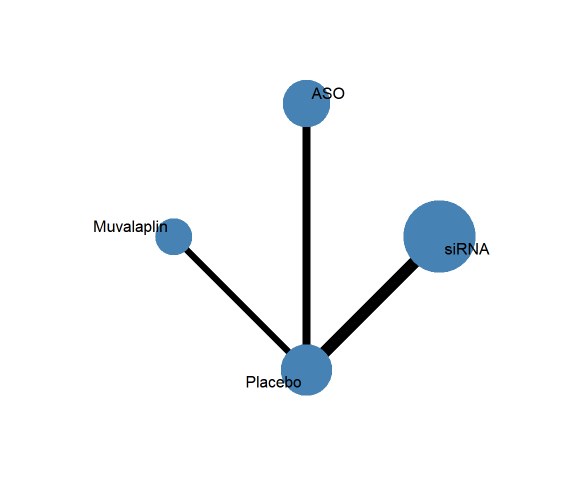


**Figure S5.14**: Network map of Injection site reactions by drug class


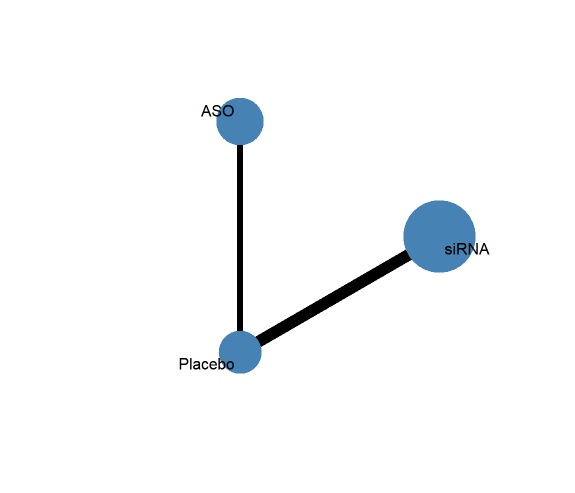


# Supplementray Material 6: Funnel plots

**Figure S6.1**: Funnel plot of Percentage Reduction in Lp(a)


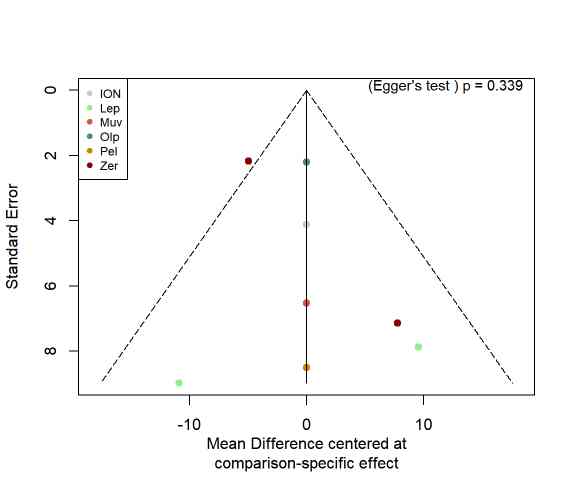


**Figure S6.2**: Funnel plot of absolute reduction in Lp(a)


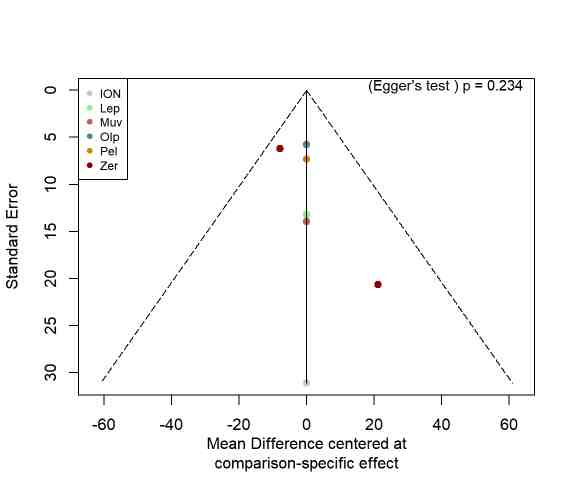


**Figure S6.3**: Funnel plot of LDL-C


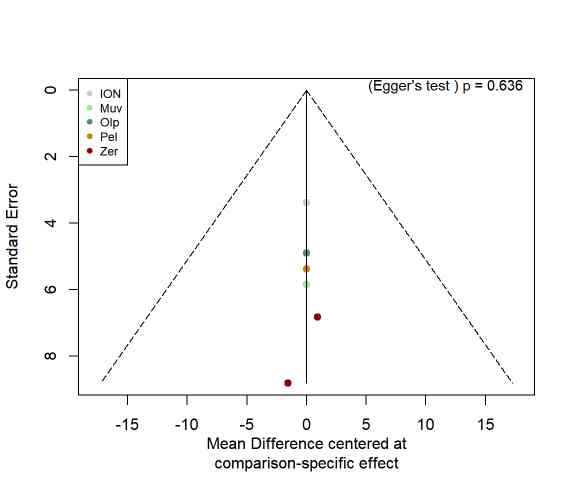


**Figure S6.4**: Funnel plot of ApoB


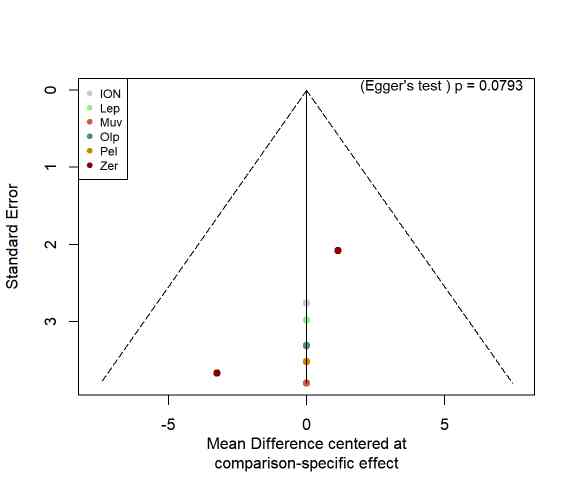


**Figure S6.5**: Funnel plot of AEs


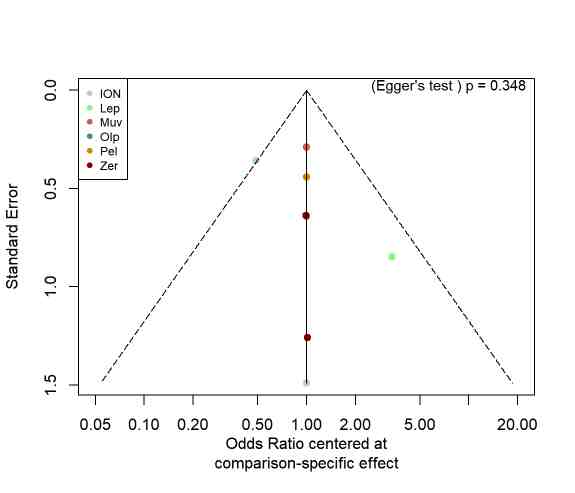


**Figure S6.6**: Funnel plot of SAEs


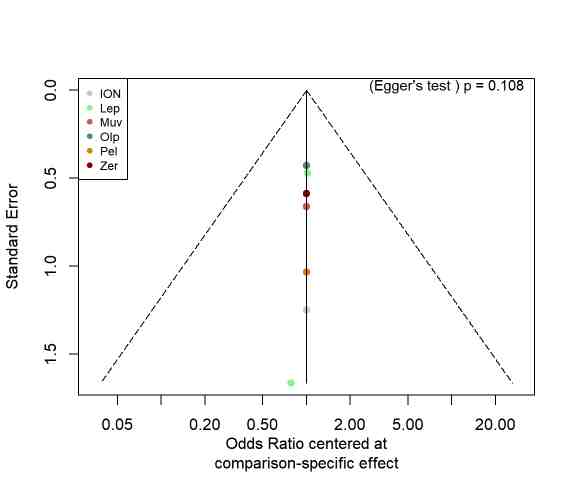


**Figure S6.7**: Funnel plot of Injection site reactions


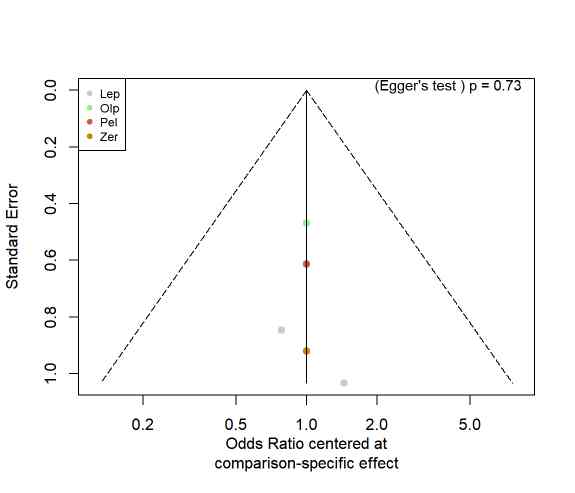


**Figure S6.8**: Funnel plot of Lp(a) Percentage Reduction by drug class


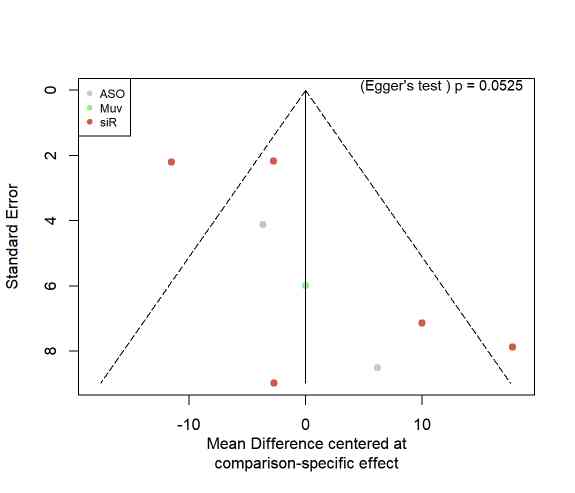


**Figure S6.9**: Funnel plot of Lp(a) Absolute Reduction by drug class


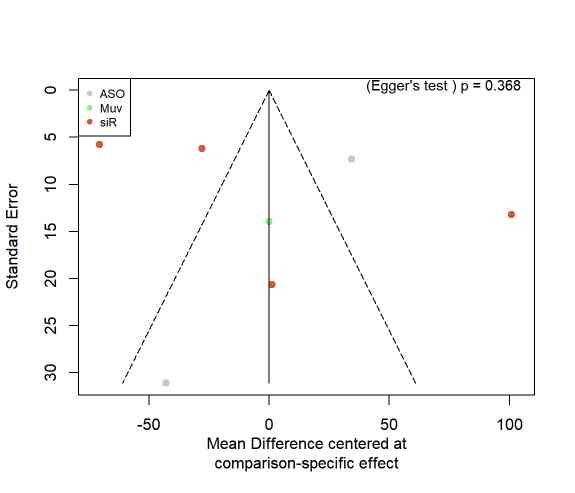


**Figure S6.10**: Funnel plot of LDL-C by drug class


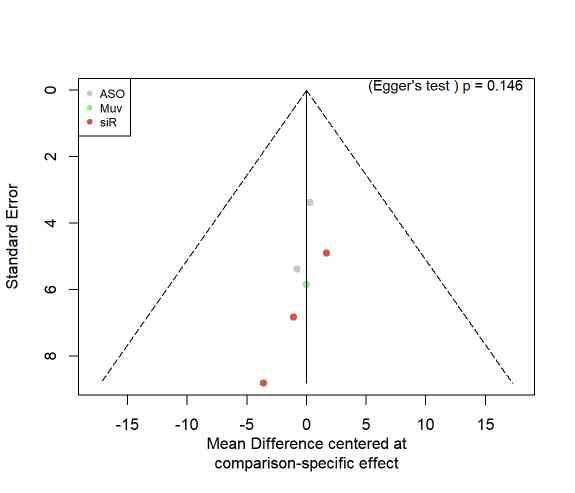


**Figure S6.11**: Funnel plot of ApoB by drug class


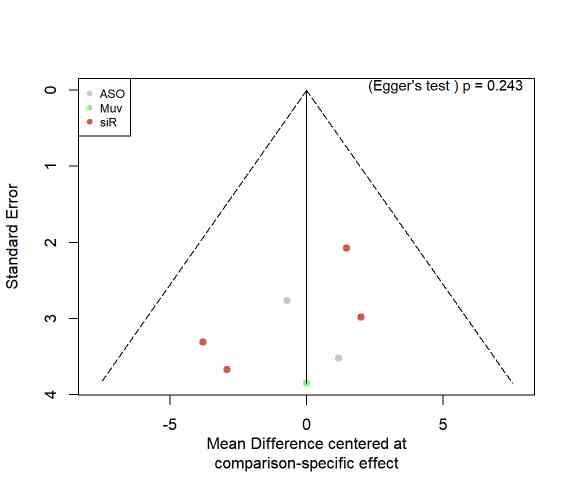


**Figure S6.12**: Funnel plot of AEs by drug class


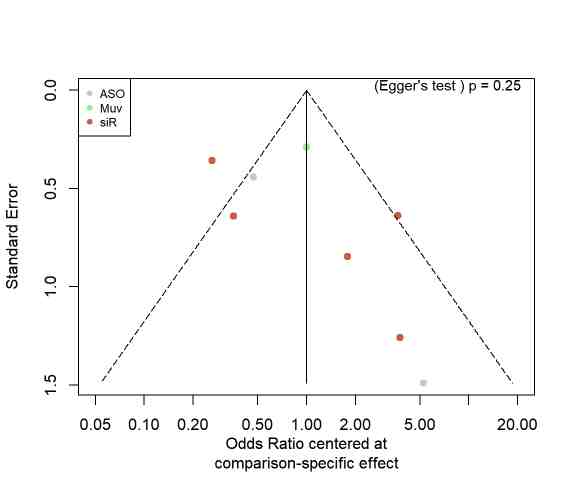


**Figure S6.13**: Funnel plot of SAEs by drug class


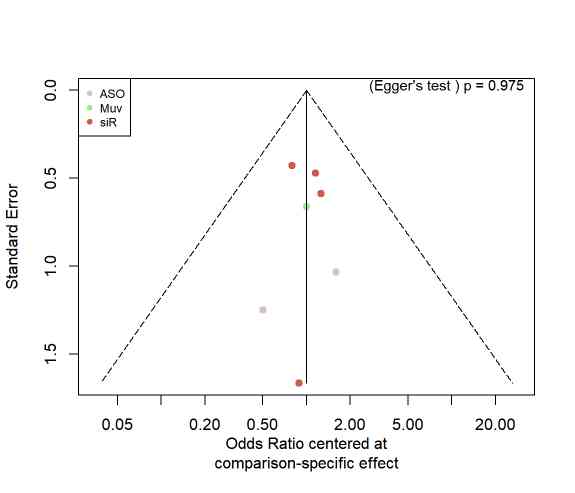


**Figure S6.14**: Funnel plot of Injection site reactions by drug class


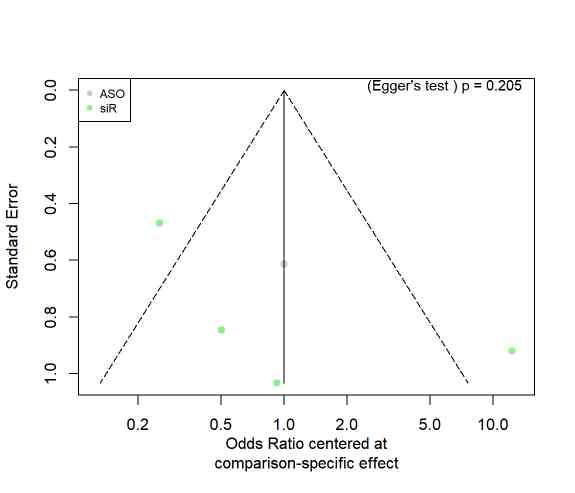


# Supplementray Material 7: forest plots of outcomes

**Figure S7.1**: Forest plot of the effect size for serious adverse events compared with placebo.


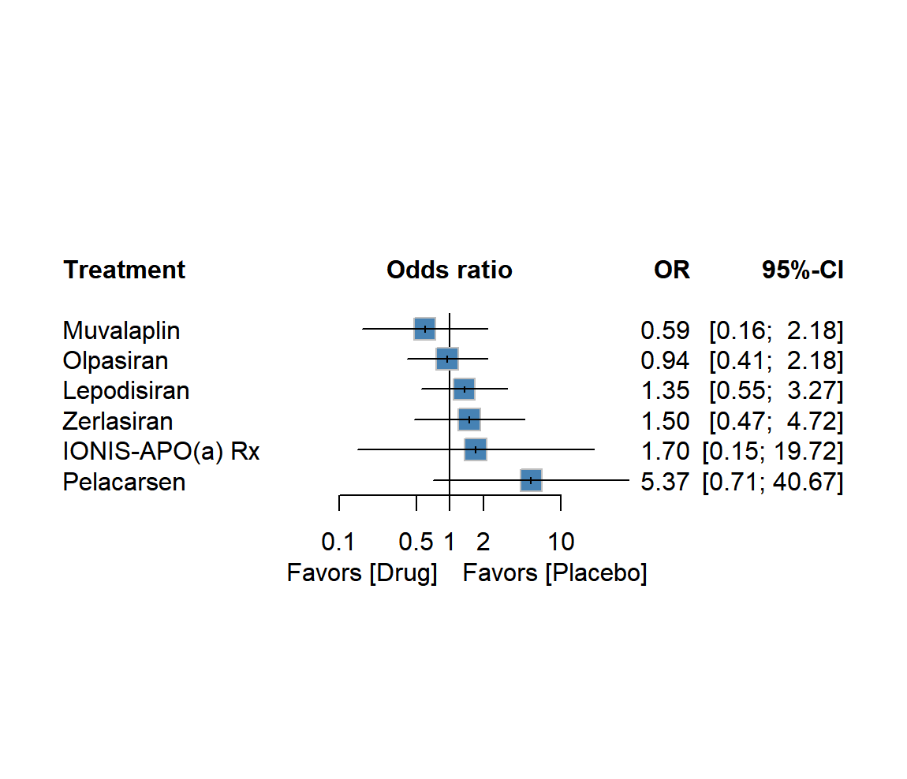


**Figure S7.2**: Forest plot of LDL-C effect sizes by drug class in subgroup analysis compared with placebo.

**
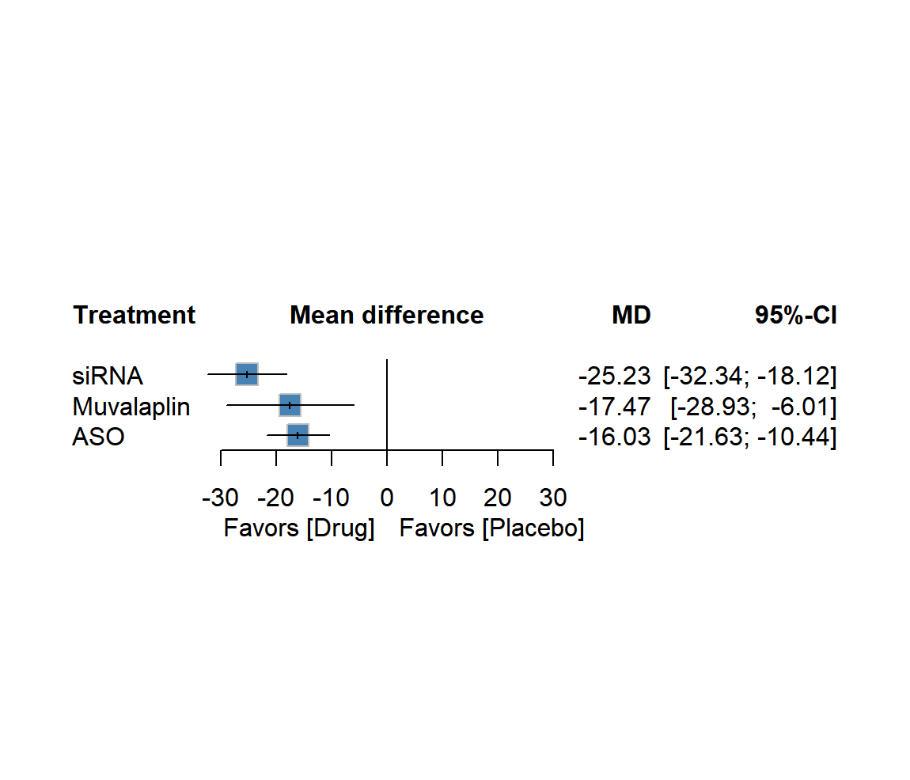
**

**Figure S7.3**: Forest plot of apoB effect sizes by drug class in subgroup analysis compared with placebo.


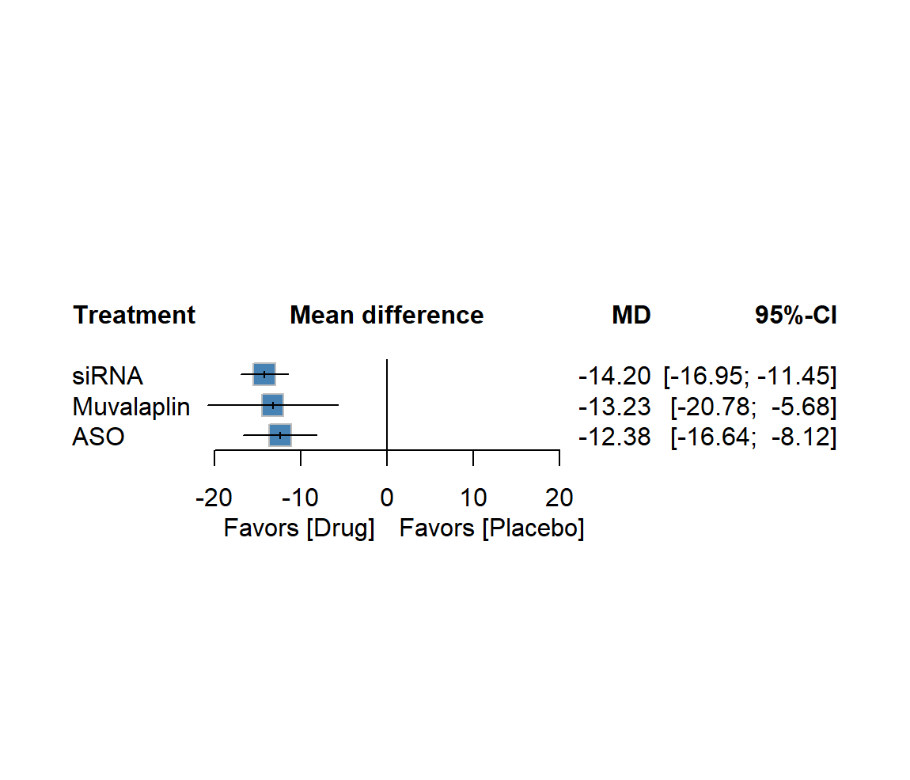


**Figure S7.4**: Forest plot of adverse events effect sizes by drug class in subgroup analysis compared with placebo.


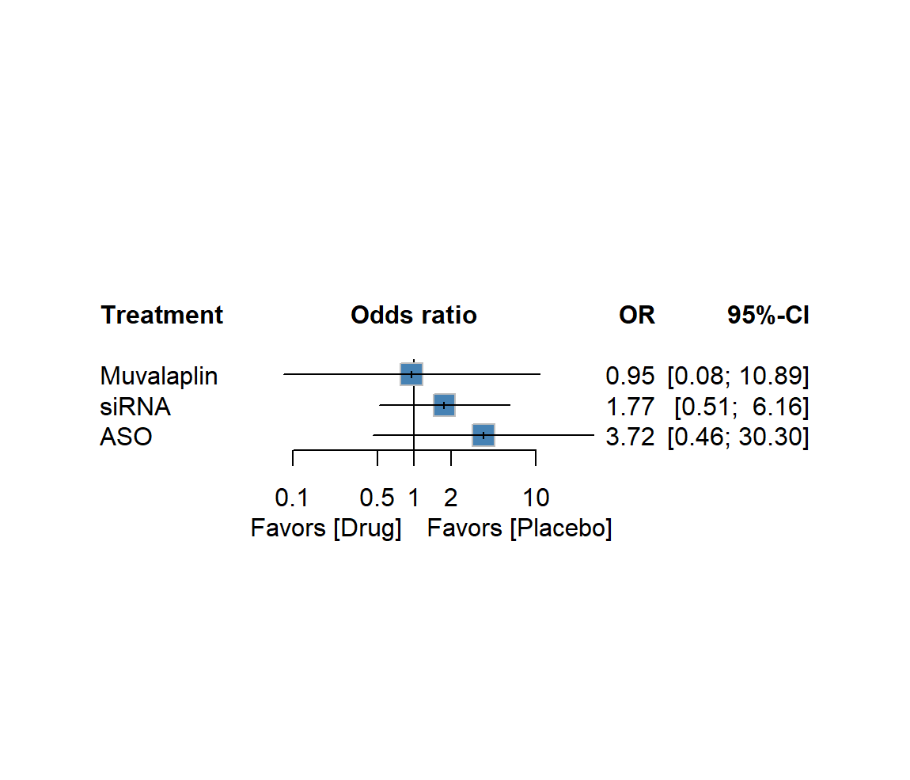


**Figure S7.5**: Forest plot of serious adverse events effect sizes by drug class in subgroup analysis compared with placebo.


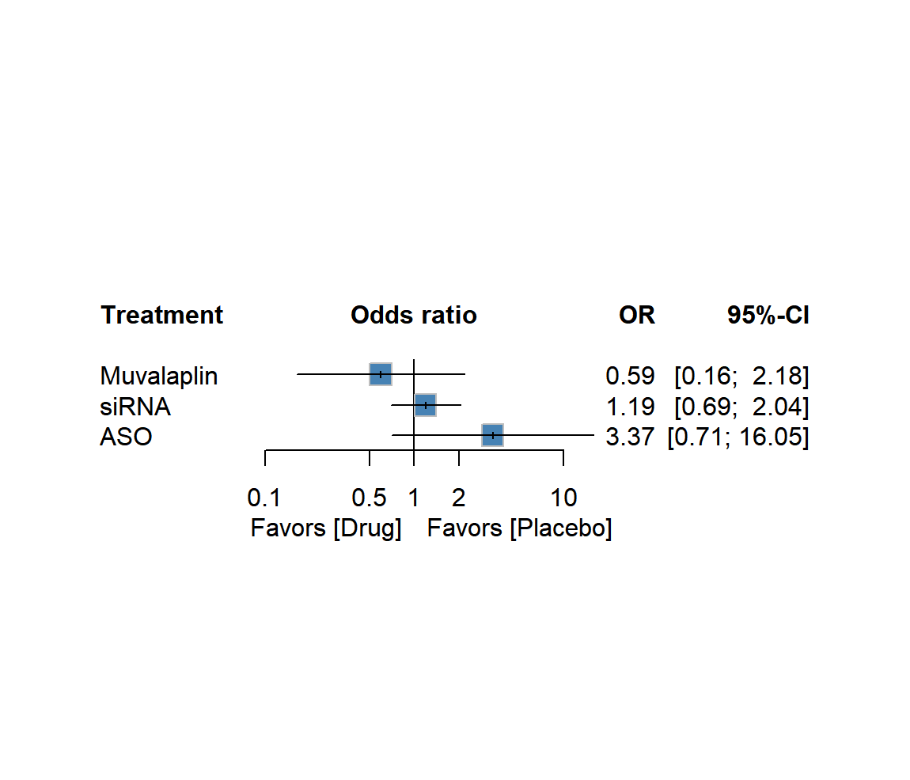


**Figure S7.6**: Forest plot of injection site reactions effect sizes by drug class in subgroup analysis compared with placebo.


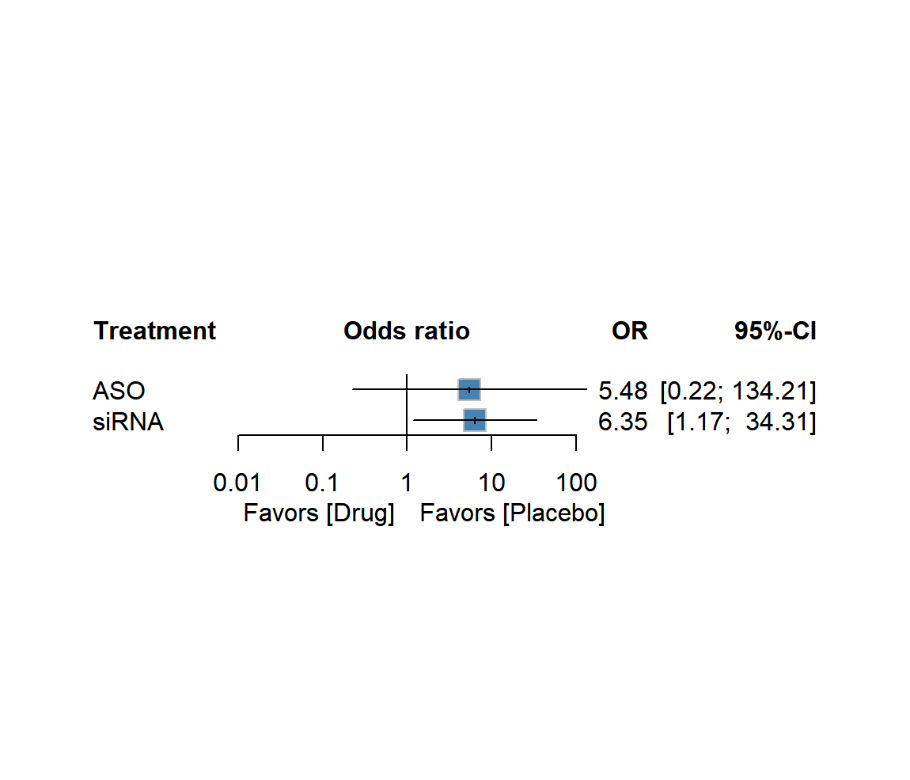


**Figure S7.7**: Forest plot of Lp(a) percentage reduction effect sizes by dose in subgroup analysis compared with placebo.


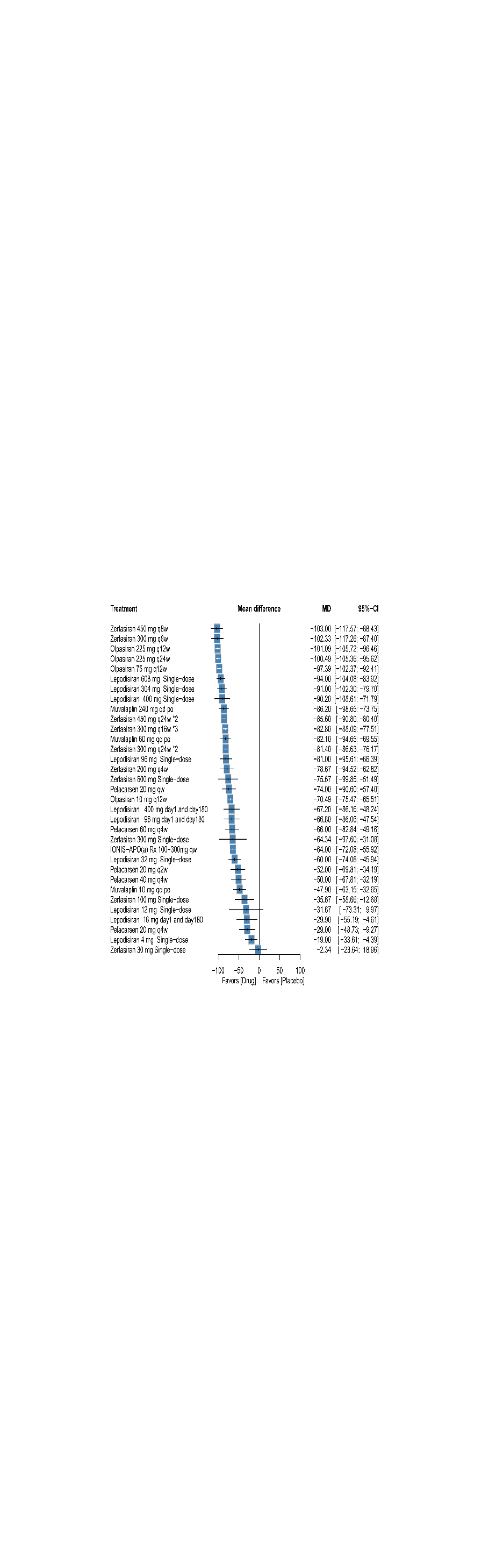


**Figure S7.8**: Forest plot of Lp(a) absolute reduction effect sizes by dose in subgroup analysis compared with placebo.


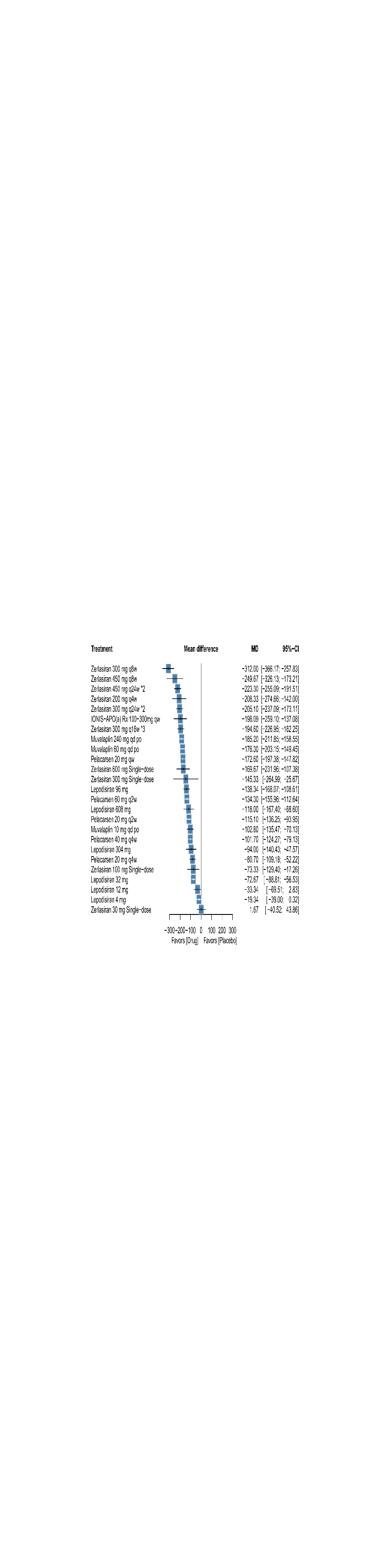


# Supplementray Material 8: League table of network meta-analysis results for Lp(a)-targeted therapeutics

The columns represent the comparison of the row drug class to the column drug class. The rows represent the comparison of the row drug class to the column drug class.

**Table S8.1:** Lp(a) Percentage Reduction

| IONIS-APO(a)Rx | . | . | . | . |  | . |
| --- | --- | --- | --- | --- | --- | --- |
| 8.38(-17.03,33.78) | Lepodisiran | . | . | . |  | . |
| 12.76(-15.90,41.42) | 4.38(-22.89,31.66) | Muvalaplin | . | . |  | . |
| 28.06(2.05,54.07) | 19.68(-4.78,44.15) | 15.30(-12.54,43.14) | Olpasiran | . |  | . |
| -9.85(-40.44,20.74) | -18.23(-47.52,11.07) | -22.61(-54.77,9.55) | -37.91(-67.73,-8.09) | Pelacarsen |  | . |
| 14.33(-9.19,37.85) | 5.95(-15.86,27.76) | 1.57(-23.96,27.10) | -13.73(-36.24,8.77) | 24.18(-3.50,51.86) | Zerlasiran |  |
| -64.00(-83.01,-44.99) | -72.38(-89.23,-55.52) | -76.76(-98.21,-55.31) | -92.06(-109.80,-74.32) | -54.15(-78.12,-30.18) | -78.33(-92.18,-64.48) | Placebo |

**Table S8.2:** Lp(a) Absolute Reduction

| IONIS-APO(a)Rx | . | . | . | . |  | . |
| --- | --- | --- | --- | --- | --- | --- |
| -118.81(-185.07,-52.55) | Lepodisiran | . | . | . |  | . |
| -33.26(-100.14,33.62) | 85.55(47.89,123.20) | Muvalaplin | . | . |  | . |
| 52.61(-9.45,114.67) | 171.42(143.20,199.64) | 85.87(56.23,115.51) | Olpasiran | . |  | . |
| -77.27(-139.95,-14.59) | 41.54(11.96,71.11) | -44.01(-74.94,-13.08) | -129.88(-148.19,-111.57) | Pelacarsen |  | . |
| 7.54(-54.56,69.65) | 126.35(98.02,154.68) | 40.80(11.06,70.55) | -45.07(-61.29,-28.84) | 84.81(66.34,103.29) | Zerlasiran |  |
| -198.09(-259.10,-137.08) | -79.28(-105.13,-53.44) | -164.83(-192.22,-137.44) | -250.70(-262.04,-239.36) | -120.82(-135.20,-106.44) | -205.63(-217.24,-194.03) | Placebo |

**Table S8.3:** LDL-C

| IONIS-APO(a)Rx | . | . | . |  | . |
| --- | --- | --- | --- | --- | --- |
| 1.74(-11.49,14.97) | Muvalaplin | . | . |  | . |
| 7.81(-3.85,19.47) | 6.07(-8.89,21.03) | Olpasiran | . |  | . |
| 1.08(-11.37,13.53) | -0.66(-16.24,14.92) | -6.73(-21.00,7.54) | Pelacarsen |  | . |
| 11.54(-0.93,24.00) | 9.80(-5.80,25.39) | 3.73(-10.56,18.01) | 10.46(-4.48,25.39) | Zerlasiran |  |
| -15.73(-22.33,-9.13) | -17.47(-28.93,-6.01) | -23.54(-33.15,-13.93) | -16.81(-27.36,-6.26) | -27.27(-37.84,-16.70) | Placebo |

**Table S8.4:** ApoB

| IONIS-APO(a)Rx | . | . | . | . |  | . |
| --- | --- | --- | --- | --- | --- | --- |
| -0.89(-8.86,7.08) | Lepodisiran | . | . | . |  | . |
| 0.13(-9.08,9.34) | 1.02(-8.45,10.49) | Muvalaplin | . | . |  | . |
| 4.89(-3.56,13.34) | 5.78(-2.95,14.51) | 4.76(-5.12,14.64) | Olpasiran | . |  | . |
| -1.89(-10.67,6.89) | -1.00(-10.05,8.05) | -2.02(-12.18,8.14) | -6.78(-16.26,2.70) | Pelacarsen |  | . |
| 0.70(-5.77,7.17) | 1.59(-5.25,8.43) | 0.57(-7.68,8.82) | -4.19(-11.58,3.21) | 2.59(-5.17,10.35) | Zerlasiran |  |
| -13.10(-18.52,-7.68) | -12.21(-18.06,-6.36) | -13.23(-20.68,-5.78) | -17.99(-24.48,-11.50) | -11.21(-18.12,-4.30) | -13.80(-17.35,-10.26) | Placebo |

**Table S8.5:** AEs

| IONIS-APO(a)Rx | . | . | . | . |  | . |
| --- | --- | --- | --- | --- | --- | --- |
| 20.79(0.54,799.90) | Lepodisiran | . | . | . |  | . |
| 20.71(0.47,915.12) | 1.00(0.10,9.53) | Muvalaplin | . | . |  | . |
| 31.26(0.60,1623.28) | 1.50(0.12,18.68) | 1.51(0.10,22.83) | Olpasiran | . |  | . |
| 11.18(0.24,522.60) | 0.54(0.05,5.64) | 0.54(0.04,6.99) | 0.36(0.02,5.85) | Pelacarsen |  | . |
| 2.99(0.07,129.32) | 0.14(0.02,1.33) | 0.14(0.01,1.66) | 0.10(0.01,1.40) | 0.27(0.02,3.35) | Zerlasiran |  |
| 19.64(0.68,565.16) | 0.94(0.23,3.93) | 0.95(0.16,5.46) | 0.63(0.08,5.01) | 1.76(0.27,11.38) | 6.56(1.2,36.00) | Placebo |

**Table S8.6:** SAEs

| IONIS-APO(a)Rx | . | . | . | . |  | . |
| --- | --- | --- | --- | --- | --- | --- |
| 1.26(0.09,17.13) | Lepodisiran | . | . | . |  | . |
| 2.86(0.18,45.80) | 2.26(0.47,10.91) | Muvalaplin | . | . |  | . |
| 1.80(0.13,24.02) | 1.43(0.42,4.84) | 0.63(0.13,2.95) | Olpasiran | . |  | . |
| 0.32(0.01,7.59) | 0.25(0.03,2.28) | 0.11(0.01,1.22) | 0.18(0.02,1.57) | Pelacarsen |  | . |
| 1.13(0.08,17.04) | 0.90(0.21,3.85) | 0.40(0.07,2.25) | 0.63(0.15,2.62) | 3.59(0.35,36.86) | Zerlasiran |  |
| 1.70(0.15,19.72) | 1.35(0.55,3.27) | 0.59(0.16,2.18) | 0.94(0.41,2.18) | 5.37(0.71,40.67) | 1.50(0.47,4.72) | Placebo |

**Table S8.7:**Injection site reactions

| Lepodisiran | . | . |  | . |
| --- | --- | --- | --- | --- |
| 2.53(0.52,12.29) | Olpasiran | . |  | . |
| 0.74(0.13,4.32) | 0.29(0.06,1.33) | Pelacarsen |  | . |
| 0.05(0.01,0.48) | 0.02(0.00,0.16) | 0.07(0.01,0.61) | Zerlasiran |  |
| 4.07(1.13,14.71) | 1.61(0.64,4.03) | 5.48(1.64,18.26) | 78.00(12.83,474.05) | Placebo |

**Table S8.8:** Lp(a) Percentage Reduction by drug class

| ASO | . |  | . |
| --- | --- | --- | --- |
| 16.41(-7.99,40.81) | Muvalaplin |  | . |
| 20.20(3.97,36.43) | 3.79(-18.00,25.58) | siRNA |  |
| -60.35(-74.20,-46.50) | -76.76(-96.84,-56.68) | -80.55(-89.01,-72.09) | Placebo |

**Table S8.9:** Lp(a) Absolute Reduction by drug class

| ASO | . |  | . |
| --- | --- | --- | --- |
| 9.60(-141.33,160.54) | Muvalaplin |  | . |
| 24.96(-82.80,132.73) | 15.36(-120.75,151.47) | siRNA |  |
| -155.23(-244.30,-66.15) | -164.83(-286.68,-42.98) | -180.19(-240.85,-119.53) | Placebo |

**Table S8.10:** LDL-C by drug class

| ASO | . |  | . |
| --- | --- | --- | --- |
| 1.44(-11.32,14.19) | Muvalaplin |  | . |
| 9.19(0.14,18.24) | 7.76(-5.73,21.25) | siRNA |  |
| -16.03(-21.63,-10.44) | -17.47(-28.93,-6.01) | -25.23(-32.34,-18.12) | Placebo |

**Table S8.11:** ApoB by drug class

| ASO | . |  | . |
| --- | --- | --- | --- |
| 0.85(-7.82,9.52) | Muvalaplin |  | . |
| 1.82(-3.25,6.89) | 0.97(-7.06,9.00) | siRNA |  |
| -12.38(-16.64,-8.12) | -13.23(-20.78,-5.68) | -14.20(-16.95,-11.45) | Placebo |

**Table S8.12:** AEs by drug class

| ASO | . |  | . |
| --- | --- | --- | --- |
| 3.92(0.16,98.00) | Muvalaplin |  | . |
| 2.10(0.18,24.05) | 0.53(0.03,8.29) | siRNA |  |
| 3.72(0.46,30.30) | 0.95(0.08,10.89) | 1.77(0.51;6.16) | Placebo |

**Table S8.13:** SAEs by drug class

| ASO | . |  | . |
| --- | --- | --- | --- |
| 5.67(0.74,43.18) | Muvalaplin |  | . |
| 2.83(0.54,14.77) | 0.50(0.12,2.04) | siRNA |  |
| 3.37(0.71,16.05) | 0.59(0.16,2.18) | 1.19(0.69,2.04) | Placebo |

**Table S8.14:** Injection site reactions by drug class

| ASO |  | . |
| --- | --- | --- |
| 0.86(0.02,32.11) | siRNA |  |
| 5.48(0.22,134.21) | 6.35(1.17,34.31) | Placebo |

# Supplementray Material 9: P-scores of the effects of various Lp(a)-targeted therapeutics

**Table S9.1:** P-scores of the effects of various Lp(a)-targeted therapeutics

| **Lp(a) Percentage** | | **Lp(a) absolute Reduction** | | **LDL-C** | | **Apo-B** | |
| --- | --- | --- | --- | --- | --- | --- | --- |
| **treatment** | **p-score** | **treatment** | **p-score** | **treatment** | **p-score** | **treatment** | **p-score** |
| Olpasiran | 0.9437 | Olpasiran | 0.9920 | Zerlasiran | 0.8933 | Olpasiran | 0.8980 |
| Zerlasiran | 0.7013 | Zerlasiran | 0.7651 | Olpasiran | 0.7638 | Zerlasiran | 0.6152 |
| Muvalaplin | 0.6568 | IONIS-APO(a) Rx | 0.7136 | Muvalaplin | 0.4911 | Muvalaplin | 0.5607 |
| Lepodisiran | 0.56 | Muvalaplin | 0.5276 | Pelacarsen | 0.4592 | IONIS-APO(a) Rx | 0.5472 |
| IONIS-APO(a)Rx | 0.3867 | Pelacarsen | 0.3346 | IONIS-APO(a) Rx | 0.3920 | Lepodisiran | 0.4728 |
| Pelacarsen | 0.2515 | Lepodisiran | 0.1672 | Placebo | 0.0005 | Pelacarsen | 0.4059 |
| Placebo | 0.0000 | Placebo | 0.0000 |  |  | Placebo | 0.0002 |
|  |  |  |  |  |  |  |  |
| **AE** | | **SAE** | | **injection-site reaction** | |  |  |
| **treatment** | **p-score** | **treatment** | **p-score** | **treatment** | **p-score** |  |  |
| Olpasiran | 0.7647 | Muvalaplin | 0.8227 | Placebo | 0.9563 |  |  |
| Lepodisiran | 0.6684 | Olpasiran | 0.6495 | Olpasiran | 0.7438 |  |  |
| Muvalaplin | 0.6614 | Placebo | 0.6286 | Lepodisiran | 0.4413 |  |  |
| Placebo | 0.6569 | Lepodisiran | 0.4519 | Pelacarsen | 0.3554 |  |  |
| Pelacarsen | 0.4786 | IONIS-APO(a) Rx | 0.4250 | Zerlasiran | 0.0032 |  |  |
| Zerlasiran | 0.1719 | Zerlasiran | 0.4161 |  |  |  |  |
| IONIS-APO(a) Rx | 0.0981 | Pelacarsen | 0.1062 |  |  |  |  |

**Table S9.2:** P-scores of the effects of various Lp(a)-targeted therapeutics by drug class

| **Lp(a) Percentage Reduction** | | **Lp(a) absolute Reduction** | | **LDL-C** | | **Apo-B** | |
| --- | --- | --- | --- | --- | --- | --- | --- |
| **treatment** | **p-score** | **treatment** | **p-score** | **treatment** | **p-score** | **treatment** | **p-score** |
| siRNA | 0.8754 | siRNA | 0.7542 | siRNA | 0.9490 | siRNA | 0.7843 |
| Muvalaplin | 0.7576 | Muvalaplin | 0.6527 | Muvalaplin | 0.5719 | Muvalaplin | 0.6607 |
| ASO | 0.3670 | ASO | 0.5917 | ASO | 0.4787 | ASO | 0.5549 |
| Placebo | 0.0000 | Placebo | 0.0014 | Placebo | 0.0005 | Placebo | 0.0001 |
|  |  |  |  |  |  |  |  |
| **AE** | | **SAE** | | **injection-site reaction** | |  |  |
| **treatment** | **p-score** | **treatment** | **p-score** | **treatment** | **p-score** |  |  |
| Placebo | 0.7297 | Muvalaplin | 0.8567 |  |  |  |  |
| Muvalaplin | 0.6623 | Placebo | 0.6295 |  |  |  |  |
| siRNA | 0.4117 | siRNA | 0.4407 |  |  |  |  |
| ASO | 0.1963 | ASO | 0.0730 |  |  |  |  |

# Supplementray Material 10: CINeMA Results

**Table S10.1**: CINeMA Results of Lp(a) Percentage Reduction

| Comparison | Within-study bias | Reporting bias | Indirectness | Imprecision | Heterogeneity | Incoherence | Confidence rating |
| --- | --- | --- | --- | --- | --- | --- | --- |
| IONIS-APO(a) Rx:Lepodisiran | No concerns | Low risk | No concerns | No concerns | No concerns | Some concerns | Moderate |
| IONIS-APO(a) Rx:Muvalaplin | No concerns | Low risk | No concerns | No concerns | Some concerns | Some concerns | Low |
| IONIS-APO(a) Rx:Olpasiran | No concerns | Low risk | No concerns | No concerns | No concerns | Some concerns | Moderate |
| IONIS-APO(a) Rx:Zerlasiran | No concerns | Low risk | No concerns | No concerns | Some concerns | Some concerns | Low |
| Muvalaplin:Lepodisiran | No concerns | Low risk | No concerns | No concerns | No concerns | Some concerns | Moderate |
| Muvalaplin:Olpasiran | No concerns | Low risk | No concerns | No concerns | Some concerns | Some concerns | Low |
| Muvalaplin:Zerlasiran | No concerns | Low risk | No concerns | No concerns | No concerns | Some concerns | Moderate |
| Pelacarsen:IONIS-APO(a) Rx | No concerns | Low risk | No concerns | No concerns | Some concerns | Some concerns | Low |
| Pelacarsen:Lepodisiran | No concerns | Low risk | No concerns | Some concerns | No concerns | Some concerns | Low |
| Pelacarsen:Muvalaplin | No concerns | Low risk | No concerns | No concerns | Some concerns | Some concerns | Low |
| Pelacarsen:Olpasiran | No concerns | Low risk | No concerns | No concerns | No concerns | Some concerns | Moderate |
| Pelacarsen:Zerlasiran | No concerns | Low risk | No concerns | No concerns | No concerns | Some concerns | Moderate |
| Placebo:IONIS-APO(a) Rx | No concerns | Low risk | Some concerns | No concerns | No concerns | Some concerns | Low |
| Placebo:Lepodisiran | No concerns | Low risk | No concerns | No concerns | No concerns | Some concerns | Moderate |
| Placebo:Muvalaplin | No concerns | Low risk | No concerns | No concerns | No concerns | Some concerns | Moderate |
| Placebo:Olpasiran | No concerns | Low risk | No concerns | No concerns | No concerns | Some concerns | Moderate |
| Placebo:Pelacarsen | Some concerns | Low risk | No concerns | No concerns | No concerns | Some concerns | Low |
| Placebo:Zerlasiran | No concerns | Low risk | No concerns | No concerns | No concerns | Some concerns | Moderate |
| Zerlasiran:Lepodisiran | No concerns | Low risk | No concerns | No concerns | No concerns | Some concerns | Moderate |
| Zerlasiran:Olpasiran | No concerns | Low risk | No concerns | No concerns | No concerns | Some concerns | Moderate |

**Table S10.2:** CINeMA Results of LDL-C

| Comparison | Within-study bias | Reporting bias | Indirectness | Imprecision | Heterogeneity | Incoherence | Confidence rating |
| --- | --- | --- | --- | --- | --- | --- | --- |
|  |  |  |  |  |  |  |  |
| IONIS-APO(a) Rx:Muvalaplin | No concerns | Low risk | No concerns | No concerns | Major concerns | Some concerns | Very low |
| IONIS-APO(a) Rx:Olpasiran | No concerns | Low risk | No concerns | Some concerns | No concerns | Some concerns | Low |
| IONIS-APO(a) Rx:Zerlasiran | No concerns | Low risk | Some concerns | Some concerns | No concerns | Some concerns | Very low |
| Muvalaplin:Olpasiran | No concerns | Low risk | No concerns | Some concerns | Some concerns | Some concerns | Very low |
| Muvalaplin:Zerlasiran | No concerns | Low risk | No concerns | Some concerns | No concerns | Some concerns | Low |
| Pelacarsen:IONIS-APO(a) Rx | No concerns | Low risk | No concerns | No concerns | Major concerns | Some concerns | Very low |
| Pelacarsen:Muvalaplin | No concerns | Low risk | No concerns | No concerns | Major concerns | Some concerns | Very low |
| Pelacarsen:Olpasiran | No concerns | Low risk | No concerns | Some concerns | Some concerns | Some concerns | Very low |
| Pelacarsen:Zerlasiran | No concerns | Low risk | No concerns | Some concerns | No concerns | Some concerns | Low |
| Placebo:IONIS-APO(a) Rx | No concerns | Low risk | Some concerns | Some concerns | No concerns | Some concerns | Very low |
| Placebo:Muvalaplin | No concerns | Low risk | No concerns | Some concerns | No concerns | Some concerns | Low |
| Placebo:Olpasiran | No concerns | Low risk | No concerns | No concerns | Some concerns | Some concerns | Low |
| Placebo:Pelacarsen | Some concerns | Low risk | No concerns | Some concerns | No concerns | Some concerns | Very low |
| Placebo:Zerlasiran | No concerns | Low risk | Some concerns | No concerns | No concerns | Some concerns | Low |
| Plozasiran :Olpasiran | No concerns | Low risk | No concerns | No concerns | Some concerns | Some concerns | Low |
| Zerlasiran:Olpasiran | No concerns | Low risk | No concerns | No concerns | Major concerns | Some concerns | Very low |

**Table S10.3:** CINeMA Results of Apo-B

| Comparison | Within-study bias | Reporting bias | Indirectness | Imprecision | Heterogeneity | Incoherence | Confidence rating |
| --- | --- | --- | --- | --- | --- | --- | --- |
|  |  |  |  |  |  |  |  |
| Placebo:Zerlasiran | No concerns | Low risk | No concerns | No concerns | Some concerns | Some concerns | Low |
| Placebo:Lepodisiran | No concerns | Low risk | No concerns | No concerns | Some concerns | Some concerns | Low |
| Placebo:Olpasiran | No concerns | Low risk | No concerns | No concerns | Some concerns | Some concerns | Low |
| Placebo:Pelacarsen | Some concerns | Low risk | No concerns | Some concerns | No concerns | Some concerns | Very low |
| Placebo:IONIS-APO(a) Rx | No concerns | Low risk | Some concerns | No concerns | Some concerns | Some concerns | Very low |
| Placebo:Muvalaplin | No concerns | Low risk | No concerns | Some concerns | No concerns | Some concerns | Low |
| Zerlasiran:Lepodisiran | No concerns | Low risk | No concerns | No concerns | Some concerns | Some concerns | Low |
| Zerlasiran:Olpasiran | No concerns | Low risk | No concerns | No concerns | Some concerns | Some concerns | Low |
| Pelacarsen:Zerlasiran | No concerns | Low risk | No concerns | No concerns | Some concerns | Some concerns | Low |
| IONIS-APO(a) Rx:Zerlasiran | No concerns | Low risk | Some concerns | No concerns | Some concerns | Some concerns | Very low |
| Muvalaplin:Zerlasiran | No concerns | Low risk | No concerns | No concerns | Some concerns | Some concerns | Low |
| Lepodisiran :Olpasiran | No concerns | Low risk | No concerns | Some concerns | No concerns | Some concerns | Low |
| Pelacarsen:Lepodisiran | No concerns | Low risk | No concerns | No concerns | Major concerns | Some concerns | Very low |
| IONIS-APO(a) Rx:Lepodisiran | No concerns | Low risk | No concerns | No concerns | Major concerns | Some concerns | Very low |
| Muvalaplin:Lepodisiran | No concerns | Low risk | No concerns | No concerns | Some concerns | Some concerns | Low |
| Pelacarsen:Olpasiran | No concerns | Low risk | No concerns | Some concerns | No concerns | Some concerns | Low |
| IONIS-APO(a) Rx:Olpasiran | No concerns | Low risk | No concerns | Some concerns | No concerns | Some concerns | Low |
| Muvalaplin:Olpasiran | No concerns | Low risk | No concerns | No concerns | No concerns | Some concerns | Moderate |
| Pelacarsen:IONIS-APO(a) Rx | No concerns | Low risk | No concerns | No concerns | Major concerns | Some concerns | Very low |
| Pelacarsen:Muvalaplin | No concerns | Low risk | No concerns | Some concerns | No concerns | Some concerns | Low |
| IONIS-APO(a) Rx:Muvalaplin | No concerns | Low risk | No concerns | No concerns | Some concerns | Some concerns | Low |
